# Molecular Adaptations and Quality Enhancements in a Hybrid (*Erythroculter ilishaeformis* ♀ × *Ancherythroculter nigrocauda* ♂) Cultured in Saline–Alkali Water

**DOI:** 10.3390/biology14060718

**Published:** 2025-06-18

**Authors:** Lang Zhang, Qiuying Qin, Qing Li, Yali Yu, Ziwei Song, Li He, Yanhong Sun, Liting Ye, Guiying Wang, Jing Xu

**Affiliations:** 1 Yangtze River Fisheries Research Institute, Chinese Academy of Fishery Sciences, Wuhan 430223, China; zhanglang@yfi.ac.cn (L.Z.); ylyu8811@yfi.ac.cn (Y.Y.); 2021203010012@whu.edu.cn (Z.S.); heli@yfi.ac.cn (L.H.); 2024308120018@webmail.hzau.edu.cn (L.Y.); 2 College of Pharmacy, South Central University for Nationalities, Wuhan 430074, China; 2022120658@mail.scuec.edu.cn; 3 Fisheries Research Institute, Wuhan Academy of Agricultural Sciences, Wuhan 430207, China; liqing@wuhanagri.com (Q.L.); sunyanhong@wuhanagri.com (Y.S.); 4 Wuhan Xianfeng Aquaculture Technology Co., Ltd., Wuhan 430207, China

**Keywords:** saline–alkali stress, antioxidant defense, immune response, environment, nutrition

## Abstract

Declining freshwater resources have spurred interest in saline–alkali (SA) water aquaculture, with species like tilapia and rainbow trout demonstrating ecological plasticity in such environments. However, the molecular mechanisms underlying fish adaptation and quality impacts remain unclear. This study investigated the hybrid fish “Xianfeng No. 1” (*Erythroculter ilishaeformis* × *Ancherythroculter nigrocauda*), a key aquaculture species in China, under 60-day SA exposure. The results showed increased levels of oxidative stress markers (MDA) and antioxidant enzymes (SOD, CAT, GSH-Px), alongside improved quality traits. Transcriptomics revealed differentially expressed genes (DEGs) in muscle tissue associated with oxidative stress (*UQCRFS1*, *UQCR10*, *CYC1*), ion transport (*COX5A*, *COX7C*, *COX7B*), and the immune response (*ATG9A*, *ATG2B*, *ATG2A*, *ULK1*, *ULK2*, *CFI, CFH*). Metabolomics identified increased non-volatile flavors (e.g., glycine, proline) and collagen-related compounds. Integrated analysis highlighted the upregulation of *GSR* and *GGT*, and the downregulation of *CHDH* and *GBSA*, potentially driving glycine accumulation. These findings suggest that SA stress enhances antioxidant capacity, activates immune pathways, and modulates ion transport, enabling adaptation while improving meat quality. This study elucidates molecular mechanisms of fish acclimation to SA environments, providing insights for sustainable aquaculture development and breeding of stress-tolerant species in SA regions.

## Simple Summary

Global freshwater shortages are pushing farmers to explore fish farming in “saline–alkali” water, but how fish adapt to these conditions and whether their meat quality changes remains unclear. To investigate this phenomenon, we conducted a study on a hybrid fish reared in saline–alkali water for a duration of two months, employing enzyme activity assays and multi-omics analysis. The findings revealed that the fish developed stress defense mechanisms conferring protection against adverse environmental conditions. This suggests the potential for employing saline–alkali water in sustainable aquaculture practices, especially in regions where freshwater resources are limited.

## 1. Introduction

Saline–alkali (SA) water is a widespread phenomenon globally, typically forming in regions experiencing prolonged dry spells, inadequate rainfall, or intense evaporation [1]. In China alone, approximately 46 million hectares of such water exist [2]. The consequences of excessive concentrations of SA are severe, disrupting the growth, survival, and reproduction of aquatic life in water bodies such as rivers and lakes. This disruption leads to reduced biomass and significant shifts in ecological diversity [3,4]. Moreover, contemporary research suggests that climate change exacerbates the salinization and alkalization of water ecosystems, posing a threat to the already limited habitat available for freshwater aquaculture [5]. This trend has heightened concerns regarding the increasing salinity and alkalinity of water environments, prompting efforts to enhance aquaculture in such challenging conditions. Moreover, there is evidence indicating negative impacts on physiological functions such as immune modulation and oxidative stress [6]. For instance, alkaline stress triggers reactive oxygen species (ROS)-mediated activation of the mitogen-activated protein kinase (MAPK) pathway and mammalian target of rapamycin (mTOR) inhibition, leading to autophagy in *Eriocheir sinensis* [7]. Additionally, alkaline conditions can induce physiological disorders in fish, such as hypocapnia and respiratory alkalosis [8]. Furthermore, under low-salinity conditions, fish must allocate more energy to osmotic pressure regulation and less energy to growth, disrupting biochemical homeostasis and causing growth retardation [9]. Thus, understanding the intricate molecular processes governing fish responses to SA stress could significantly mitigate these impacts and enhance the efficient utilization of SA waters for fish farming.

Saline waters not only influence the adaptation and survival of fish but also have an impact on their essential nutritional components and quality attributes [10]. For instance, research has shown that low-salt conditions significantly alter the metabolic pathways of amino acids and fatty acids in fish, resulting in noticeable changes in flavor and meat quality [11,12]. SA stress can modify the biochemical composition of fish muscle, thereby affecting its nutritional and sensory properties, which are critical factors for consumer preference and market value [13]. These alterations may manifest as fluctuations in the levels of free amino acids, contributing to taste, and modifications in fatty acid profiles, influencing both the health benefits and taste of fish [11]. While existing studies have addressed the impact of SA stress on amino acids, fatty acids, and traditional nutritional components of fish, there is still a gap in understanding its effects on the muscle quality characteristics of fish.

The ongoing progression of omics techniques has significantly advanced the field of systems biology and provided indispensable tools for investigating aquatic organisms [14,15,16]. The recent research focus has shifted towards utilizing transcriptomics to unravel the adaptive mechanisms employed by fish in SA environments [9,17]. Previously, we conducted transcriptomic studies on Xiangfeng No. 1, investigating its transcriptomic expression under various experimental conditions [18]. Transcriptomics reveals gene expression patterns but lacks resolution for metabolite-driven pathway analysis [19,20]. In contrast, metabolomics has emerged as a critical technique for elucidating the intricate association between metabolite fluxes and the internal or external stimuli encountered by organisms, offering new avenues for in-depth research in this domain [21]. The use of a single omics technology often poses challenges in comprehensively deciphering the underlying molecular mechanisms of environmental stress responses. In comparison, multi-omics approaches are valuable tools for comprehensively and precisely evaluating mechanisms in the field [22,23]. Therefore, the synergistic integration of transcriptomics and metabolomics analyses holds promise for providing a nuanced understanding of the interplay between metabolic pathways and gene expression networks, revealing the intricate coordination involved.

The top-mouth culter (*Erythroculter ilishaeformis*) is a highly consumed commercial fish in China [24]. However, artificial breeding of this species can be challenging and costly due to its substantial size. In contrast, *Ancherythroculter nigrocauda*, a member of the Cyprinidae family, is smaller in size and of lower economic significance due to its slower rate of development [25]. In recent years, scientists have successfully utilized distant hybridization techniques to create a hybrid F1 known as “Xianfeng No. 1” for aquaculture purposes [26]. Offspring resulting from the cross between *A. nigrocauda* (♂) and *E. ilishaeformis* (♀) exhibit superior traits, including increased stress tolerance, compared to their parental species, along with enhanced disease resistance [27]. Moreover, these hybrid fish are easier to catch and transport and are well-suited to aquaculture in ponds. To advance the expansion of hybrid aquaculture into SA environments, it is critical to investigate the mechanistic basis of its adaptability in SA stress and systematically characterize the physiological and genetic traits that enable its survival in such conditions. Regrettably, there exists a dearth of research regarding the tolerance of the species to SA waters and the potential methods for enhancing the nutritional value of its fish fillets [28].

In this research, we conducted a thorough study using multi-omics techniques to analyze gene expression and metabolic changes in the hybrid fish “Xianfeng No. 1” exposed to SA conditions. Our goal was to identify the molecular mechanisms involved in the fish’s response and adaptation to this stress. Furthermore, our study evaluated alterations in the quality traits of hybrids in SA water through non-targeted metabolomics analysis. The findings of this investigation have the potential to enhance our knowledge of stress responses in fish, contribute to the advancement of aquaculture practices, and aid in the development of breeding strategies for species with increased tolerance to SA conditions.

## 2. Materials and Methods

### 2.1. Ethics Statement

The culter hybrids are widely cultivated in mainland China and are not subject to protection. All experiments conducted adhered to the guidelines of the Yangtze River Fisheries Research Institute, Chinese Academy of Fishery Sciences (Permit No. 2022zhanglang002).

### 2.2. Chemical Reagents

The chemical reagents NaHCO_3_ (Cat No. 10018960) and NaCl (Cat No. 10019318) were purchased from the National Medicines Corporation Ltd., Beijing, China.

### 2.3. Experimental Animals and Design

The hybrid fish were acclimatized in an indoor recirculating water system with predefined water parameters for a duration of two weeks before the initiation of the experiment. Following the acclimation period, 120 hybrid fish with an average weight of 162.21 ± 32.41 g and a body length of 25.6 ± 2.3 cm were randomly selected and divided into two groups (*n* = 60). Subsequently, each group was allocated to six separate circular flumes with specific dimensions (height = 80 cm; diameter = 140 cm); each flume contained 10 hybrid fish. One group was designated as the control group and provided with fresh water, while the other group was subjected to SA stress, achieved by adding NaHCO_3_ (12 mmol/L) and NaCl (86 mmol/L) to the water. The concentrations of SA were determined based on data from previous studies [29]. After an additional three-day acclimation period without feeding, the formal experiment was initiated.

The fish were provided with commercial pellets (Haida Co., Ltd., Nantong City, Jiangsu Province, China) that were formulated in accordance with the Chinese national standard for fish feed (GB/T 22919.5-2008) [30]. The feed composition included 32% crude protein, 6% lipid, 4% crude fiber, 12% ash, and 40% carbohydrate, supplemented with trace amounts of vitamins and minerals to fulfill nutritional requirements. Feeding was conducted twice daily until the fish exhibited visible satisfaction. Every three days, one-third of the water in the circular flumes was replaced. Over the course of 60 days, water quality parameters were monitored daily (pH 8.5 ± 0.5; dissolved oxygen > 6.8 mg/L; NH3 0–0.4 mg/L; nitrite 0–0.2 mg/L). Notably, no fish mortality occurred throughout the duration of the experiment.

### 2.4. Measurement of Enzyme Activities

The activities of the enzymes malondialdehyde (MDA), superoxide dismutase (SOD), catalase (CAT), and glutathione peroxidase (GSH-Px) were assessed utilizing specific assay kits provided by Jiancheng Bioengineering (MDA: Cat. No. A003-1-2, SOD: Cat. No. A001-3, CAT: Cat. No. A007-1, GSH-Px: Cat. No. A005-1-2). Muscle tissue samples were homogenized in ice-cold physiological saline at a 1:9 weight-to-volume ratio, followed by centrifugation at 2500× *g* at 4 °C for 10 min to obtain the supernatant for subsequent analysis. Protein concentrations in the supernatant were quantified using the bicinchoninic acid (BCA) assay, as per the manufacturer’s protocol (Jiancheng Bioengineering Testing Kit, Nanjing, China, Cat. No. A045-4-2). Absorbance was measured at 562 nm using a Shimadzu UV-1800 spectrophotometer, Kyoto, Japan, and protein concentrations were calculated based on a standard curve generated from bovine serum albumin (BSA). The assays for enzymatic activities and subsequent calculations for MDA, SOD, CAT, and GSH-Px were performed in accordance with the manufacturer’s guidelines and established literature [31,32], with results normalized to protein concentration.

### 2.5. Sample Collection

On the 60th day of the experiment, before sampling, all 120 fish were anesthetized using MS-222 (100 mg/L, Sigma, St. Louis, MO, USA). Their muscle tissues were then dissected on ice. The tissues were then immediately frozen in liquid nitrogen and stored at stored at −80 °C to ensure experimental stability for the quantitative polymerase chain reaction (qRT-PCR) and RNA sequencing (RNA-seq). All sampling procedures were conducted within an ultra-clean workstation. Sixty muscle samples were collected from each group. For transcriptomics analysis, every five muscles were combined to create one sample, with three randomly selected samples per group. For ultra-high-pressure liquid chromatography–mass spectrometry (UHPLC-MS) metabolomics analysis, every five remaining muscles were combined to create one sample, resulting in six samples per group.

### 2.6. Metabolomics Analysis

The samples were thawed at 4 °C and then divided into aliquots, which were added to a cold solution containing methanol, acetonitrile, and water in a 2:2:1 (*v*/*v*) ratio. The mixture was vortexed, sonicated for 30 min, and centrifuged at 14,000× *g* for 20 min at 4 °C. The resulting supernatant was collected, dried under vacuum conditions, and reconstituted using a solution of acetonitrile and water in a 1:1 volume ratio. Then, the samples were analyzed using mass spectrometry.

The raw mass spectrometry data contained within wiff.scan files were converted into MzXML files using ProteoWizard MSConvert (Version 3.0.20069) and imported into XCMS software (Version 3.14.0) for analysis. CAMERA was used for the annotation of isotopes and adducts. Ion features were retained when at least one group exhibited more than 50% non-zero measurements. Metabolite identification was accomplished using known accurate *m*/*z* values (<10 ppm) and MS/MS with an in-house database that includes readily accessible authentic standards (Personal Bio, Shanghai, China).

For data analysis, the R package ropls was utilized (v4.1.2; R Core Team, Vienna, Austria, 2021), and multivariate data analysis was conducted using Pareto-scaled principal component analysis (PCA) and orthogonal partial least squares discriminant analysis (OPLS-DA) to visualize metabolic differences between the control and experimental groups. Variables with a Variable Importance in Projection (VIP) > 1 and *p*-value < 0.05 were considered statistically significant. Metabolite-based pathway analysis was performed using MetaboAnalyst 5.0 (http://www.metaboanalyst.ca (accessed on 1 December 2023)) with the Kyoto Encyclopedia of Genes and Genomes (KEGG) pathway database (http://www.genome.jp/kegg/ (accessed on 1 December 2023)).

### 2.7. Transcriptomics Analysis

#### 2.7.1. RNA Extraction and Sequencing

RNA extraction from control and treated samples was conducted using TRIzol reagent (Invitrogen, Waltham, MA, USA), following the manufacturer’s protocol with phase separation by chloroform. RNA precipitation was achieved through isopropanol incubation and subsequent centrifugation at 12,000× *g* for 15 min at 4 °C, followed by two ethanol (75%) washes. The concentration and purity of RNA were assessed based on the ratios of A260/A280 and 28S/18S. Only samples with RNA integrity numbers (RINs) exceeding 7.0 were included in subsequent analyses. RNA-seq was performed by Shanghai Paisennuo Biotechnology Co., Ltd., Shanghai, China, and the sequencing of the cDNA libraries was performed using the Illumina HiSeqTM 4000 platform, which yielded 150 bp paired-end reads. Adapter trimming and quality filtering were performed using Trimmomatic v0.39.

#### 2.7.2. Transcriptome Annotation

Trinity software version 2.4.0 was used to conduct de novo transcriptome assembly with clean reads, as previously described [33]. Gene function annotation was performed using several databases, including NCBI non-redundant protein sequences (NR), Kyoto Encyclopedia of Genes and Genomes (KEGG), Gene Ontology (GO), evolutionary genealogy of genes Non-supervised Orthologous Groups (eggNOG), protein family (Pfam), and Swissprot databases; details are given in Table S1.

#### 2.7.3. Transcriptome Assembly

The RNA-seq data was analyzed as previously described [18]. Quality control measures were implemented using the Trimgalore v0.4.3 software to remove adapter sequences (https://github.com/FelixKrueger/TrimGalore (accessed on 12 December 2023)). The fastq files were assessed with FASTQC v0.11.5 (http://www.bioinformatics.babraham.ac.uk/projects/fastqc/ (accessed on 12 December 2023)), and a perl script was utilized to clean the raw fastq data by removing low-quality sequences, adaptor sequences, and poly-N sequences, resulting in clean reads. Next, an analysis was conducted to determine the Q20, Q30, and GC content, as well as sequence duplication levels, before submission to the National Center for Biotechnology Information (NCBI) with the ID PRJNA1097380 (https://dataview.ncbi.nlm.nih.gov/object/PRJNA1097380 (accessed on 8 April 2024)).

In accordance with previous studies [34], differentially expressed genes (DEGs) were identified using stringent criteria (adjusted *p*-value < 0.05 and |log2 (Fold Change)| ≥1). R software (v4.1.2; R Core Team, 2021) was used for data analysis and construct volcano plots and heatmaps. KOBAS software (version 2.1.1) was utilized to assess the statistical significance of DEG enrichment based on KEGG pathway enrichment and Gene Ontology (GO) analysis using a false discovery rate (FDR) threshold of <0.05 to determine statistically significant enrichments.

### 2.8. Quantitative Real-Time PCR Analysis

Table S2 presents primer sequences for genes related to the oxidation–reduction process, which were utilized to validate the RNA-seq data. In line with prior research, *β*-*actin* was selected as the internal reference gene for the hybrid species under investigation [27]. Furthermore, the stability of β-actin expression was confirmed under the experimental conditions, justifying its continued use as an internal control gene. Prior to total RNA extraction, DNase I was employed to eliminate DNA, following procedures described in a previous study [18]. RT-qPCR was conducted using SYBR Green. The relative gene expression was determined utilizing the 2^−△△Ct^ method [35].

### 2.9. Construction of PPI Network

Protein–protein interaction (PPI) networks were constructed using STRING v12.0 to analyze functional associations among genes linked to specific Gene Ontology (GO) terms, focusing on oxidative stress, ion transport, the complement system, and autophagy in the hybrid under SA stress. Hub genes were identified within each GO category based on maximal interaction connectivity. Network visualization was performed using Cytoscape (Version 3.10.1) to elucidate interaction patterns and key molecular pathways.

### 2.10. Statistical Analysis

Data analysis was performed using the Statistical Package for the Social Sciences (SPSS) version 15.0. Independent Student’s *t*-tests were used to compare differences between the control and saline–alkali groups, with significance levels set at *p*-values less than 0.05. The results are presented as the mean ± standard deviation (S.D.), with asterisks (*) denoting statistically significant differences between the experimental and control groups (* *p* < 0.05, ** *p* < 0.01, and *** *p* < 0.001).

## 3. Results

### 3.1. Effect of SA Exposure on Biochemical Indicators

**Figure 1:**
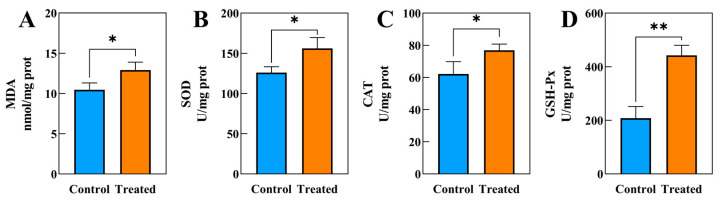
The biochemical indicators changes in muscle tissue in hybrids under SA exposure (*n* = 3). (**A**) MDA, (**B**) SOD, (**C**) CAT, (**D**) GSH-Px, * indicates *p* < 0.05, ** indicates *p* < 0.01.

After 60 days of exposure to SA water, the activities of MDA (Figure 1A), SOD (Figure 1B), CAT (Figure 1C), and GSH-Px (Figure 1D) in the SA stress group were significantly elevated compared to those in the control group.

### 3.2. Metabolome Quality Analysis

**Figure 2:**
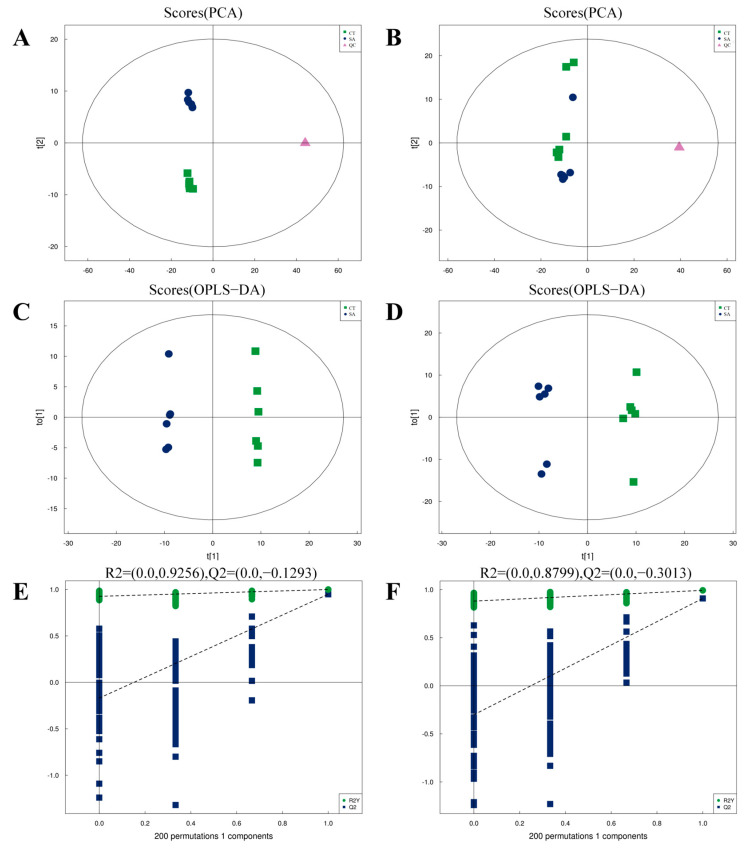
Quality analysis of metabolomics data (*n* = 6). (**A**) PCA scores plot for positive mode samples. (**B**) PCA scores plot for negative mode samples. (**C**) OPLS-DA score plot for positive mode. (**D**) OPLS-DA score plot for negative mode. (**E**) OPLS-DA permutation test for positive mode. (**F**) OPLS-DA permutation test for negative mode.

Multivariate statistical analyses, including PCA and OPLS-DA, were conducted to assess the differences in metabolites in the muscles of experimental fish. PCA was utilized to assess the inter- and intra-experimental group variability in a general sense. In both positive mode (Figure 2A) and negative mode (Figure 2B), the CT and SA groups were distinctly separated in the unsupervised principal component analysis model, suggesting that the instrument exhibited high stability and reproducibility, as evidenced by the tight clustering of quality control (QC) samples. OPLS-DA was performed to filter out noise unrelated to categorical information, thereby enhancing the model’s parsing ability and effectiveness. As shown in Figure 2C,D, the distinct separation between the CT and SA groups indicates SA’s significant impact on the metabolic profiles of the experimental fish’s muscle tissue. Furthermore, statistical graphics were produced using R software version 4.1.2 (R Core Team, 2021). It was observed that the R^2^Y and Q^2^ values in both positive and negative modes, obtained through random permutations, exceeded those of the original OPLS-DA model (Figure 2E,F). Thus, the OPLS-DA model demonstrated an excellent fit and high predictability, rendering it suitable for further analysis.

### 3.3. Differential Metabolite Identification

**Figure 3:**
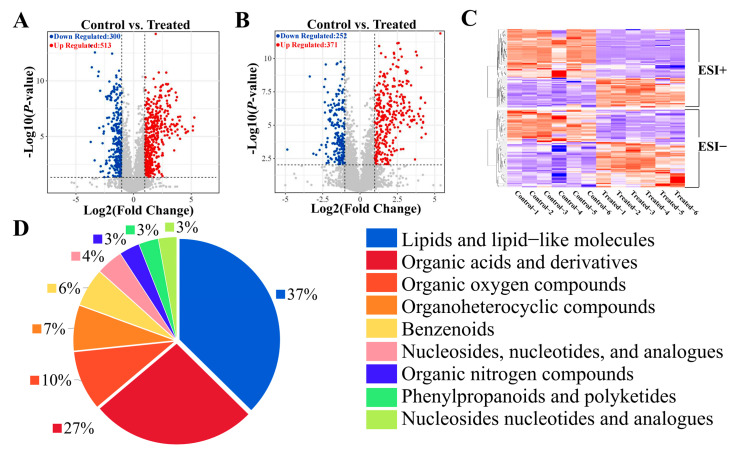
Hierarchical clustering analysis and classification of DMs. (**A**) Volcano plots comparing DMs in positive mode between CT and SA groups. The horizontal black dotted line represents the threshold of *p* = 0.05, while the vertical black dotted lines represent the threshold of |log2 (Fold Change)| = 1. (**B**) Volcano plots comparing DMs in negative mode between CT and SA groups. (**C**) Hierarchical clustering analysis of DMs in positive and negative mode, with red indicating upregulation and blue indicating downregulation. (**D**) Classification pie chart of DMs.

Next, differential metabolites (DMs) between the SA and CT groups were identified using criteria of VIP > 1 and *p*-value < 0.05. Two volcano plots were generated: Figure 3A displays a total of 813 DMs in positive mode (513 upregulated, 300 downregulated), and Figure 3B shows 623 DMs in negative mode (371 upregulated, 252 downregulated). The results of cluster analysis heatmaps, in both positive and negative modes, demonstrated that SA significantly influenced the metabolism of muscle tissues in the experimental fish (Figure 3C). Among these metabolites, the top three categories were lipids and lipid-like molecules, organic acids and derivatives, and organic oxygen compounds. Notably, lipid metabolites comprised the largest proportion (Figure 3D).

### 3.4. Pathway Enrichment and Categorization of DMs

**Figure 4:**
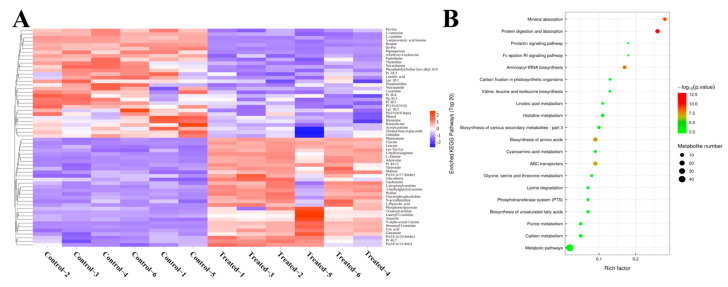
Heat map of DMs and KEGG bubble plots. (**A**) Heatmap of cluster analysis displaying the most significant DMs. (**B**) Bubble plot illustrating KEGG pathway enrichment.

To further explore the composition and interconnections of DMs and elucidate the metabolic mechanisms underlying the impact of SA on muscle tissues in experimental fish between CT and SA, a cluster analysis was performed. Figure 4A and Table S3 illustrate the most significant metabolites in the top 30 positive and negative ion patterns, revealing notable differences in substances such as amino acids and fatty acids between the SA and CT groups.

Using KEGG enrichment analysis, we determined the significance of metabolite enrichment in each pathway (Figure 4B). The results showed that the enriched pathways included mineral absorption, protein digestion and absorption, aminoacyl-tRNA biosynthesis, ABC transporters, and biosynthesis of amino acids. These results point to enhanced adaptations in nutrient uptake, protein synthesis and metabolism, and the transport of substances across membranes in experimental fish in response to environmental changes.

**Figure 5:**
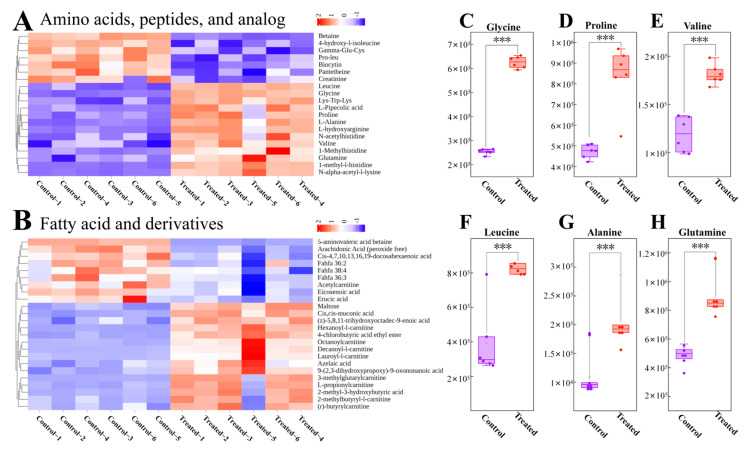
Heatmap of cluster analysis of different classes of DMs and boxplots of special DMs. (**A**) Heatmap displaying cluster analysis results for amino acids, peptides, and analogs. (**B**) Heatmap illustrating cluster analysis results for fatty acids and derivatives. (**C**–**H**) Box plots depicting specific DMs. The purple box represents the control group, while the red box represents the treated group. Each data point represents to the peak area of an individual sample. *** indicates *p* < 0.001.

Next, we generated heatmaps to categorize DMs with significant differences. The differences between CT and SA groups for amino acids, peptides, and analogs are shown in Figure 5A, while the differences for fatty acids and derivatives are shown in Figure 5B. Among these DMs, certain amino acids and flavor substances such as glycine, proline, valine, leucine, alanine, and glutamine demonstrated increased levels in the SA group (Figure 5C–H). Overall, these findings suggest that SA may impact the metabolism of flavor and collagen-related substances. In addition, amino acids, peptides, and their analogous metabolites, as well as fatty acids and their derivative metabolites, are presented in Tables S4 and S5, respectively.

### 3.5. Overview of Transcriptomics Sequencing Results

Using the Illumina HiSeq sequencing platform, we excluded sequences with junctions at the 3′ end and removed reads with average quality scores below Q20, following which we obtained high-quality sequence data comprising 41.93 million and 46.86 million reads for the CT and SA groups, respectively. Quality analysis of the obtained data revealed that the average Q20 values for the CT and SA groups were 98.16% and 97.98%, respectively, while the average Q30 values were 94.52% and 94.16% (Table S6). Additionally, the average GC content of clean reads for the CT and SA groups was 45.04% and 44.82%, respectively (Table S7). Figures S1–S5 presented the de novo assembly unigenes’ information, including their GO, KEGG, EggNOG, and NR annotations for the hybrid species. These results confirm the high quality of our transcriptome sequencing data, ensuring the credibility of subsequent analyses. The functional annotations and classification information were detailed in the Supplementary Materials, Sections S1.1 and S1.2.

### 3.6. Identification of DEGs

**Figure 6:**
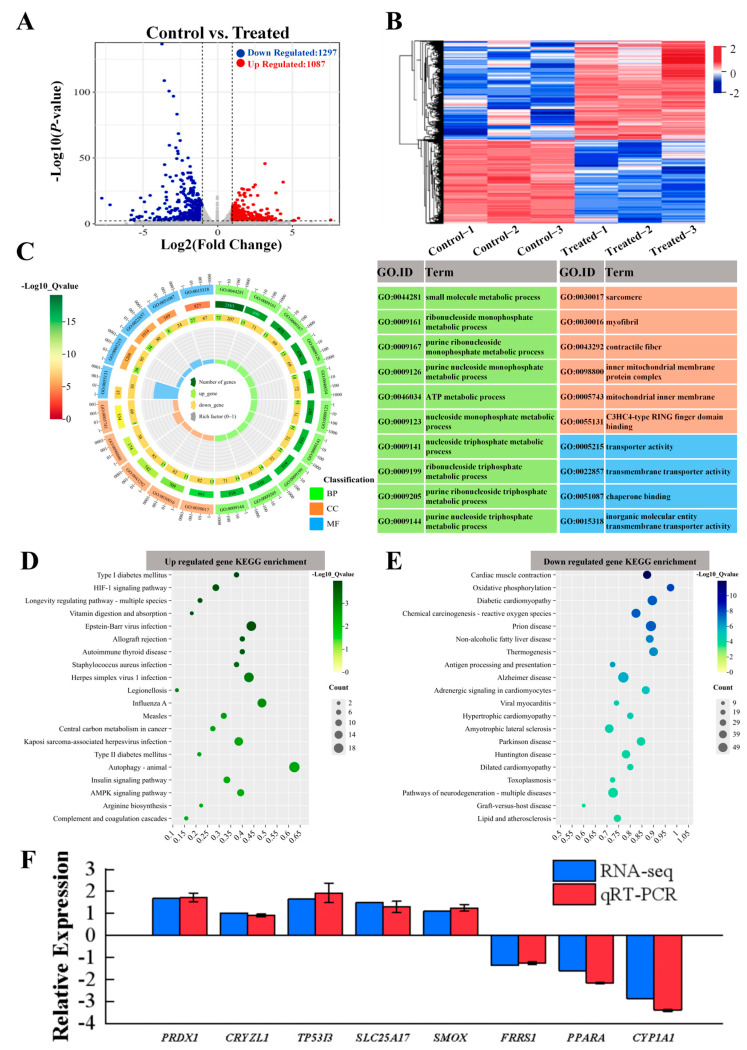
Transcriptome analysis graphs depicting differentially expressed genes (DEGs) in the muscles of hybrids under SA stress. (**A**) Volcano plots comparing DEGs between CT and SA groups. (**B**) Heatmap displaying clustering analysis results of differentially expressed genes. (**C**) Gene Ontology (GO) term enrichment analysis of significantly altered DEGs. Green corresponds to biological processes (BP), red corresponds to cellular components (CC), and blue corresponds to molecular functions (MF). A higher −log10 (Qvalue) indicates a more significant enrichment of genes. (**D**,**E**) Enrichment bubble plots illustrating up- and downregulated genes in the KEGG pathway. (**F**) Comparison of gene expression data between RNA-Seq and qRT-PCR.

Further analysis led to the identification of a total of 2384 DEGs meeting the criteria of *p*-value < 0.05 or |log2 (Fold Change)| ≥1. The overall distribution of these DEGs, with 1087 upregulated and 1297 downregulated genes is shown in Figure 6A. The heatmap of the cluster analysis of DEGs showed a significant difference in the expression of the CT versus the SA group (Figure 6B), which suggests that SA has a significant impact on cellular transcription in the muscle tissues of the experimental fish.

### 3.7. Pathway Enrichment and Verification of DEGs

Considering that DEGs have a certain biological significance, this prompted us to explore their expression patterns. Through GO enrichment analysis (Figure 6C), we found that the top biological process categories included small molecule metabolic process, ribonucleoside monophosphate metabolic process, and purine ribonucleoside monophosphate metabolic process. To further investigate the relationship between metabolic pathways and DEGs, we conducted KEGG enrichment analysis, following which we observed the upregulation of DEGs in pathways such as vitamin digestion and absorption, autophagy—animal, and Epstein–Barr virus infection (Figure 6D) and the downregulation of DEGs in pathways like cardiac muscle contraction, oxidative phosphorylation, and diabetic cardiomyopathy, suggesting potential effects of SA on immune pathways, inflammatory responses, and energy metabolism in experimental fish (Figure 6E). In addition, Tables S8–S11 presented representative DEGs associated with various biological processes: oxidation-reduction (Table S8), ion transport (Table S9), complement and coagulation cascades (Table S10), and autophagy-animals differentiation (Table S11), respectively.

**Figure 7:**
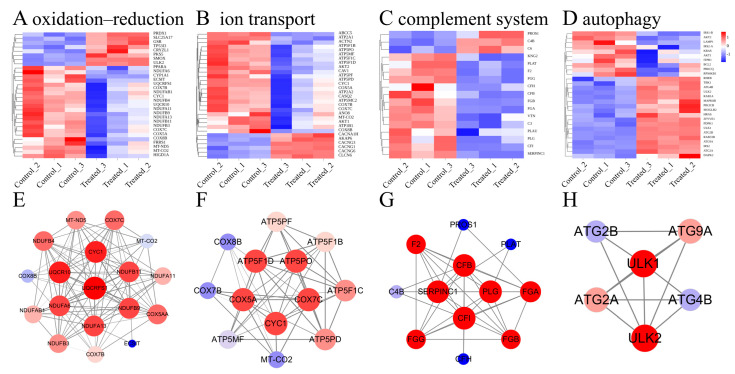
Transcriptome analysis map of differentially expressed genes (DEGs) enriched for some pathways in muscle of hybrids under SA stress. (**A**–**D**) Heatmap of some DEGs enriched for specific pathways. (**E**–**H**) PPI network diagram of some DEGs enriched for specific pathways, the redder color of the circle means that the gene is more important in that PPI network, and the thicker line means that two-by-two genes are more strongly correlated.

To validate the results of the transcriptomics analysis and to test whether SA has an effect on redox DEGs, we performed qRT-PCR on eight DEGs from the oxidation–reduction pathway. Among these, five genes were found to be upregulated (*PRDX1*, *CRYZL1*, *TP53I3*, *SLC25A17*, *SMOX*), while three genes were downregulated (*FRRS1*, *PPARA*, *CYP1A1*). The results obtained from qRT-PCR were consistent with those from RNA-Seq, indicating a similar trend in gene expression levels and reinforcing the reliability of our data (Figure 6F). As shown in Figure 7A–D, several genes associated with the oxidation–reduction, ion transport, complement system, and autophagy were identified, the PPI network diagram shows the association between some special genes (Figure 7E–H). These findings suggest that SA influences both the oxidation–reduction process and ion transport in experimental fish.

### 3.8. Metabolomics and Transcriptomics Integrated Analysis

**Figure 8:**
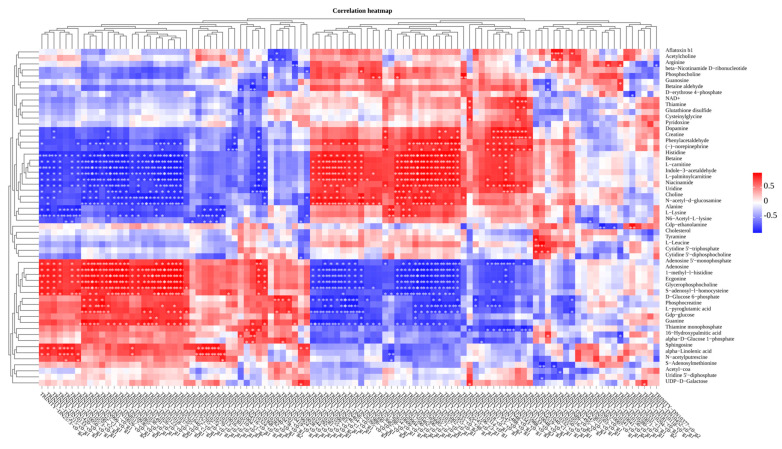
Correlation heatmap of metabolomics and transcriptomics. The heatmap illustrates the correlation between metabolomics and transcriptomics, with genes arranged in columns and metabolites quantified in behavioral units. Positive correlations are denoted in red, while negative correlations are denoted in blue. * indicates *p* < 0.05, ** indicates *p* < 0.01.

Using Pearson correlation analysis, we conducted correlation analyses between metabolomics and transcriptomics data and observed strong correlations between each transcript and metabolite in the CT and SA groups (Figure 8). For instance, a strong positive correlation (R = 0.999) was observed between the DEG symbolized as TRINITY_DN5002_c0_g1 and betaine. Conversely, choline exhibited a statistically significant inverse relationship (R = −0.984) with DEG TRINITY_DN1018_c0_g1. Both betaine and choline are involved in the synthesis of numerous amino acids, highlighting specific metabolites associated with each transcript.

**Figure 9:**
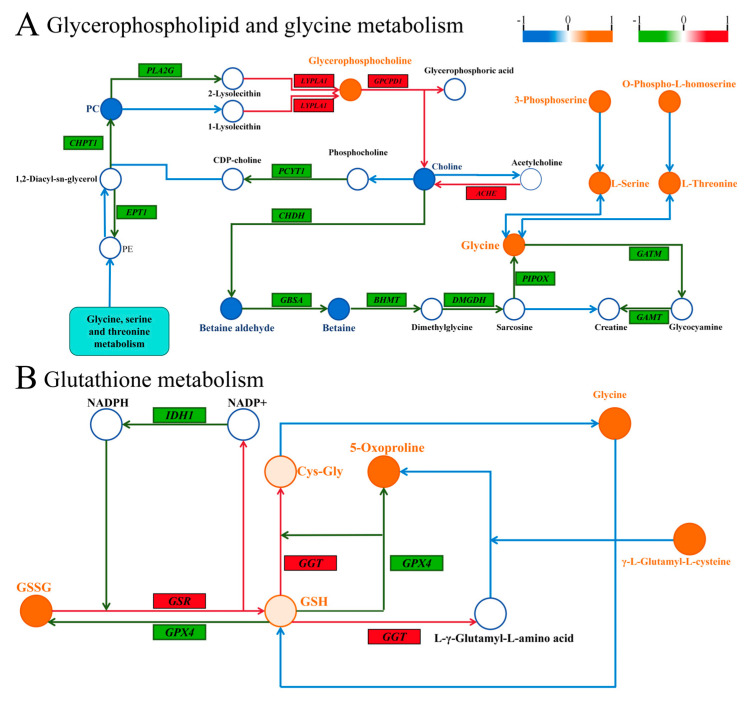
Combined metabolomics and transcriptomic citation of uncovered molecular mechanisms. (**A**) Correlation plot of genes and metabolites regulating glycerophospholipid and glycine metabolism. (**B**) Correlation plot of genes and metabolites regulating glutathione metabolism. Similarly, significant metabolite changes are depicted in orange (upregulated) or blue (downregulated), and differential genes are indicated in red (upregulated) or green (downregulated). Genes are shown in italics for clarity.

Figure 9A summarizes the relationship between DMs and DEGs in glycerophospholipid and glycine metabolism. In this context, lysophospholipase 1 (LYPLA1) was found to be upregulated, while choline dehydrogenase (CHDH) and betaine-aldehyde dehydrogenase (GBSA) were downregulated, and glycine, a DM, was upregulated. Figure 9B highlights glutathione metabolism, where glutathione-disulfide reductase (GSR) and gamma-glutamyltransferase 1 (GGT) were upregulated among the DEGs, while glutathione oxidized (GSSG) and glycine, among the DMs, were upregulated as well. These findings suggest that SA influences the expression of gene-to-metabolite networks in experimental fish muscle tissue.

## 4. Discussion

Saline–alkali (SA) water, a widespread global occurrence, remains underutilized due to its inhospitable nature for aquatic life. With freshwater sources diminishing due to climate change and overexploitation, there is increasing interest in leveraging SA water for aquaculture. This study uses a comprehensive approach, integrating metabonomics and transcriptomics, to compare data on SA stress. Our research suggests that exposure to SA water alters biochemical indicators associated with oxidative stress in fish. Furthermore, changes in gene expression related to oxidative stress, the immune response, and ion transport were detected, as well as improvements in fish quality characteristics being identified. This suggests that SA stress may boost antioxidant defense, activate immune responses, affect ion transport balance, and enhance the quality characteristics of this hybrid.

### 4.1. Influence of SA Water on Redox Metabolism

Redox homeostasis is essential for maintaining metabolism and supporting growth as it helps to eliminate excess ROS and restores cellular redox balance. However, the specific mechanism involved in redox metabolism under SA stress remains largely unknown.

Prior research has revealed that SA stress can lead to the production of ROS in aquatic animals, causing oxidative stress [7,36]. This can result in an increase in MDA concentration, which is a key factor in organism damage [37]. The accumulation of ROS in animals can lead to lipid oxidation and the formation of MDA, causing severe cell damage [38]. MDA content is a key indicator of tissue peroxidative damage. This study found higher MDA levels in the SA stress group compared to the control group, showing that long-term stress led to oxidative damage in the muscle cells of this hybrid (Figure 1A).

SOD and CAT are indispensable antioxidant enzymes in organisms [39]. The SOD-CAT system is commonly recognized as the main defense mechanism against reactive oxygen species (ROS) generation during periods of oxidative stress, playing a crucial role in scavenging reactive oxygen species and protecting cells [39]. SOD helps to convert excess O^2−^ into H_2_O_2_, which is then broken down by CAT to reduce oxidative stress [40,41]. In the current investigation, elevated levels of SOD and CAT activities were observed in the muscles of the hybrid species following 60 days of exposure to SA water (Figure 1B,C). This finding aligns with a study [42], which noted heightened SOD activity in the serum of Nile tilapia (*Oreochromis niloticus*) when subjected to elevated salinity levels. The enhanced SOD and CAT activity in the muscles of the hybrid species may be attributed to a response to SA stress, potentially serving a protective role in mitigating oxidative damage to cells. Furthermore, in addition to CAT, GSH-Px has the capability to inhibit reactive ROS production by counteracting hydrogen peroxide (H_2_O_2_) [43]. The current investigation revealed a significantly higher GSH-Px activity in the SA group compared to the CT group (Figure 1D), aligning with a recent study on the impact of SA exposure on Luciobarbus capito gills [44]. Under SA stress conditions, fish accumulate H_2_O_2_, ROS that cause oxidative damage [44]. The observed increase in GSH-Px activity likely represents a compensatory response to SA stress. Therefore, the upregulation of GSH-Px activity in this study may be linked to the removal of hydrogen peroxide buildup during SA stress.

Ubiquitin–cytochrome c oxidoreductase is a key enzyme complex on the inner mitochondrial membrane that is closely associated with oxidative phosphorylation processes [45]. As the first component of the respiratory chain, enabling the electron transfer from NADH to ubiquinone (CoQ10), the proper functioning of the electron transport chain is essential for maintaining redox balance within the cell and preventing the leakage of electrons into oxygen, which could otherwise result in the formation of ROS [46]. Proteins associated with ubiquinol–cytochrome c reductase, such as UQCRFS1, UQCR10, and CYC1, constitute integral components of the ubiquinol–cytochrome c oxidoreductase complex [47,48,49]. Significant enrichment of oxidatively phosphorylated DEGs was also detected when Nile tilapia were exposed to salt water [50]. In this study, *UQCRFS1*, *UQCR10*, and *CYC1* were significantly downregulated, suggesting that the process of oxidative phosphorylation may be inhibited under saline and alkaline stress, leading to the production of ROS (Figure 7E).

### 4.2. Influence of SA Water on Ion Transport

Ion transport plays a crucial role in regulating essential physiological parameters, including ion balance and membrane potential, which are vital for various biological functions [51]. The cytochrome c oxidase complex catalyzes the pumping of protons from the mitochondrial matrix across the inner membrane into the membrane gap along with electron transfer, a process known as proton pumping: this is the driver of oxidative phosphorylation [52]. *COX5A*, *COX7C*, and *COX7B* are subunits of the cytochrome c oxidase (COX) complex. The genes encoding ubiquitin–cytochrome c oxidoreductases are primarily associated with the redox pathway. Similarly, the genes encoding cytochrome c oxidases, discussed in the current chapter, are predominantly involved in the ion transport pathway. Ubiquitin–cytochrome c oxidoreductase and cytochrome c oxidase are critical constituents of the electron transport chain [52,53]. In high-salt environments, Na^+^ can damage mitochondria in two ways. First, excessive Na^+^ disrupts the calcium balance within mitochondria, leading to calcium buildup and the production of ROS, which directly damages the mitochondria’s energy production system [54]. Second, Na^+^ alters the mitochondrial inner membrane, hindering the free movement of essential substances like ubiquinone, which is crucial for energy production [55]. Our study demonstrated that the expression of the COX5A, COX7C, and COX7B genes was downregulated in response to saline–alkali stress. This downregulation may be mediated through two distinct mechanisms. Firstly, elevated Na^+^ concentrations compromise the structural integrity of the mitochondrial inner membrane, leading to a compensatory transcriptional downregulation aimed at mitigating the energy metabolic burden. Secondly, stress-induced excessive accumulation of reactive oxygen species (ROS) exerts feedback inhibition on gene expression through pathways such as ROS-p53 signaling, thereby effectively interrupting the self-perpetuating cycle of ROS generation.

### 4.3. Influence of SA Water on Immune Response

Autophagy is a conserved cellular recycling process critical for maintaining homeostasis, eliminating damaged components, and adapting to nutrient stress [56]. Emerging evidence underscores its interplay with immune regulation [56,57]. Central to autophagosome formation are the autophagy-related proteins *ATG2A*, *ATG2B*, and *ATG9A*. *ATG2A* collaborates with *ATG9A* to initiate autophagosome assembly [58], while *ATG2B* mediates the lipid transfer essential for autophagosome expansion and lipid droplet dynamics [59]. *ATG9A* further supports vesicle maturation through lipid remodeling [60]. Unc-51 Like Autophagy Activating Kinase 1 (*ULK1*) and Unc-51 Like Autophagy Activating Kinase 2 are two key proteins in the autophagy signaling pathway [61,62]. They regulate the formation of autophagophores by acting upstream of phosphatidylinositol 3-kinase [62,63]. In our study, we observed significant upregulation of *ATG9A*, *ATG2B*, *ATG2A*, *ULK1*, and *ULK2* (Figure 7G); this is consistent with previous studies suggesting that alkali exposure induces autophagy in fish, which was detrimental to them [7]. In the SA group, SA induces cellular autophagy, thereby impacting the immune system further.

The complement system, comprising over 35 plasma and membrane proteins, plays an important role in host defense against pathogenic microorganisms by facilitating the innate immune response and connecting innate and adaptive immunity [64]. Complement factor I (*CFI*) gene mediates the innate immune response in the yellow catfish Pelteobagrus fulvidraco [65]. Moreover, Complement Factor H (*CFH*) also contributes to maintaining a balanced immune response by regulating complement activation as a soluble inhibitor of complement. In our study [66], *CFI* and *CFH* were significantly downregulated in the SA group, suggesting that SA may impact the immune system (Figure 7H).

### 4.4. Effect of SA Water on Muscle Quality Characteristics

The chemical composition of fish meat is routinely assessed for protein, fat, moisture, amino acids, and sugar content. Collagen, the primary protein in animals, maintains the structural integrity of connective tissues like bones, skin, and cartilage, with elevated levels indicating increased muscle tone and strength [26,67]. Glycine, proline, and hydroxyproline constitute a significant portion of collagen’s amino acid composition and protein content in animals [68,69]. Valine supports amino acid transport and synthesis, promoting protein synthesis in fish [70].

Additionally, leucine and glutamine help in skeletal muscle protein synthesis, contributing to fish elasticity and tenderness [71,72,73]. In this study, glycine, proline, valine, leucine, and glutamine were found to be significantly higher in the SA group compared to the CT group, suggesting increased collagen and skeletal muscle protein levels in fish muscle tissue, which may enhance fish elasticity and palatability (Figure 5A). Sweet-tasting amino acids in fish, such as glycine, serine, and alanine, contribute to flavor perception [3,74]. Moreover, we observed that the glycine and alanine levels were higher in the SA group compared to the CT group (Figure 5A), indicating potentially sweeter-flavored fish under low-SA aquaculture conditions.

During glycerophospholipid and glycine metabolism, lysophospholipase 1 (*LYPLA1*) catalyzes the hydrolysis of lysophosphatidylcholine, yielding glycerophosphocholine (GPC) [75]. GPC is a key phospholipid constituent of cell membranes, essential for membrane stability and neurotransmitter expression [76]. Our multi-omics analysis revealed an upregulation of the *LYPLA1* gene and GPC metabolites, suggesting that saline exposure may induce *LYPLA1* gene expression, facilitating GPC synthesis and potentially enhancing cellular membrane integrity (Figure 9A). Additionally, choline undergoes oxidation by choline dehydrogenase (*CHDH*) and betaine aldehyde dehydrogenase (*GBSA*) to produce betaine, with downstream production of glycine from betaine [77,78,79]. Furthermore, we observed that the downregulation of *CHDH* and *GBSA* gene expression under SA conditions led to decreased betaine aldehyde and betaine levels and a consequent increase in glycine content downstream, suggesting that reduced upstream products may enhance downstream glycine production (Figure 9A). Meanwhile, serine is also an important source of glycine [79], which is significantly increased as shown in Figure 9A, suggesting that the increase in glycine under SA stress conditions may also result from serine conversion.

In glutathione metabolism, glutathione reductase (GSR) catalyzes the reduction of glutathione disulfide (GSSG) to glutathione (GSH), while gamma-glutamyltransferase (*GGT*) converts GSH to cysteinylglycine (Cys-Gly), a precursor substance for glycine [80]. Through the integration of transcriptomics and metabolomics, we observed a significant increase in GSSG content and elevated *GSR* expression in glutathione metabolism. Additionally, there was a notable upregulation of *GGT* expression (Figure 9B). These findings suggest that SA exposure may disrupt the balance between GSSG and glycine transformation, leading to increased glycine levels. This disturbance could affect protein metabolism and amino acid transport, potentially resulting in glycine accumulation.

In summary, our findings suggest that SA exposure could potentially increase collagen levels and enhance the content of sweet substances in fish, which may contribute to enhancing the characteristic qualities of fish, potentially influencing their taste and texture.

## 5. Conclusions

This study investigated how “Xianfeng NO.1” muscle tissues respond to prolonged saline–alkali exposure using untargeted metabolomics and transcriptomics. Saline–alkali stress significantly affected the antioxidant system, increasing levels of MDA, SOD, CAT, and GSH-Px. Genes linked to oxidative stress resistance (*UQCRFS1*, *UQCR10*, *CYC1*) and ion transport (*COX5A*, *COX7C*, *COX7B*) were upregulated, enhancing antioxidant defenses and affecting sodium and amino acid transport. Furthermore, the upregulation of immune-related genes, such as *ATG9A*, *ULK1*, *CFI*, and *CFH*, may be essential for enhancing the immune system’s efficacy. Our metabolomic analysis showed a notable rise in non-volatile flavor substances like glycine and proline under saline–alkali stress. Gene regulation in glycerophospholipids, glycine, and glutathione metabolism suggested the increased glycine production, possibly due to serine conversion. This multi-omics study offered insights into fish adaptation and muscle quality to saline–alkali environments, aiding in breeding high-quality, stress-tolerant species.

## Data Availability

Data are contained within the article and Supplementary Materials.

## Supplementary Materials

The following supporting information can be downloaded at https://www.mdpi.com/article/10.3390/biology14060718/s1, Figure S1: The information of de novo assembly unigenes. Figure S2: GO annotations of the assembled unigenes of hybrid species. Figure S3: KEGG annotations of the assembled unigenes of hybrid species. Figure S4: EggNOG annotations of the assembled unigenes of hybrid species. Figure S5: NR annotations of the assembled unigenes of hybrid species. (**A**) Distribution of species. (**B**) E-value distribution of comparisons. (**C**) Sequence similarity distribution. Table S1. Summary of annotation results. Table S2. Primers used in the quantitative PCR analysis. Table S3. Significantly different metabolites. Table S4. Amino acids, peptides, and analogue metabolites. Table S5. Fatty acid and derivatives metabolites. Table S6. Statistical assessment of sequencing data. Table S7. Overall sequence statistics. Table S8. Representative oxidation-reduction-related DEGs. Table S9. Representative ion transport-related DEGs. Table S10. Representative complement and coagulation cascades-related DEGs. Table S11. Representative autophagy-animals differentiatio-related DEGs.
