# Supplementary material for: Molecular Adaptations and Quality Enhancements in a Hybrid (Erythroculter ilishaeformis ♀ × Ancherythroculter nigrocauda ♂) Cultured in Saline–Alkali Water
Source: Biology (Basel). 2025 Jun 18;14(6):718. doi: 10.3390/biology14060718 (PMC12189855; doi:10.3390/biology14060718)
Supplement: Supplementary file 1 [file biology-14-00718-s001.zip › biology-3677952-supplementary.pdf]

# Supplementary Materials

**Supporting files:**

**Abbreviation**

**Supplemental results**

**Table S1–S6**

**Figure S1–S5**

**Abbreviation**

| Abbreviation | Full Name |
|---|---|
| ABC transporters | ATP-binding cassette transporters |
| ANOVA | Analysis of variance |
| ATG2A | Autophagy Related 2A |
| ATG2B | Autophagy Related 2B |
| ATG9A | Autophagy-Related Protein 9A |
| ATP | Adenosine triphosphate |
| BCA | Bicinchoninic acid |
| BP | Biological Process (GO category) |
| CAMERA | Collection of Algorithms for MEtabolite pRofile Annotation |
| CAT | Catalase |
| CC | Cellular Component (GO category) |
| CFH | Complement Factor H |
| CFI | Complement factor I |
| CHDH | Choline dehydrogenase |
| CoQ10 | Coenzyme Q10 / Ubiquinone |
| CT | Control group |
| CYC1 | Ubiquinol-Cytochrome-C Reductase Complex Cytochrome C1 Subunit |
| Cys-Gly | Cysteinylglycine |
| DEGs | Differentially Expressed Genes |
| DMs | Differential Metabolites |
| DO | Dissolved Oxygen |
| eggNOG | evolutionary genealogy of genes: Non-supervised Orthologous Groups |
| FDR | False Discovery Rate |
| GC content | Guanine-Cytosine content |
| GBSA | Betaine-aldehyde dehydrogenase |
| GO | Gene Ontology |
| GPC | Glycerophosphocholine |
| GSH | Glutathione (reduced) |
| GSH-Px | Glutathione Peroxidase |
| GSR | Glutathione-disulfide Reductase |
| GSSG | Glutathione oxidized / Glutathione disulfide |
| GGT | Gamma-glutamyltransferase 1 |
| KEGG | Kyoto Encyclopedia of Genes and Genomes |
| log2FC | log2 (Fold Change) |
| LYPLA1 | Lysophospholipase 1 |

| | |
|---|---|
| MAPK | Mitogen-activated protein kinase |
| MDA | Malondialdehyde |
| MF | Molecular Function (GO category) |
| MS-222 | Tricaine methanesulfonate |
| mTOR | Mammalian target of rapamycin |
| Multi-omics | Integration of multiple omics technologies (e.g., transcriptomics, metabolomics) |
| MzXML | Mass Spectrometry XML format |
| NADH | Nicotinamide adenine dinucleotide (reduced) |
| NCBI NR | National Center for Biotechnology Information Non-redundant protein sequences database |
| NEG | Negative Ion Mode (Mass Spectrometry) |
| NH3 | Ammonia |
| OPLS-DA | Orthogonal Partial Least Squares Discriminant Analysis |
| PCA | Principal Component Analysis |
| PE reads | Paired-End reads |
| Pfam | Protein family database |
| PI3K | Phosphatidylinositol 3-kinase |
| POS | Positive Ion Mode (Mass Spectrometry) |
| PPARA | Peroxisome proliferator activated receptor alpha |
| PPI | Protein-Protein Interaction |
| PRDX1 | Peroxiredoxin 1 |
| qRT-PCR | Quantitative Real-Time Polymerase Chain Reaction |
| QC | Quality Control |
| Q20 | Base call accuracy of 99% (Phred score >=20) |
| Q30 | Base call accuracy of 99.9% (Phred score >=30) |
| R | Statistical programming language/environment |
| RIN | RNA Integrity Number |
| RNA-Seq | RNA Sequencing |
| ROS | Reactive Oxygen Species |
| R2Y | Fraction of Y variation explained by the model (OPLS-DA) |

| | |
|---|---|
| Q2 | Fraction of the total variation of Y predicted by the model (OPLS-DA) |
| SA | Saline-alkali |
| S.D. | Standard Deviation |
| SLC25A17 | Solute carrier family 25 member 17 |
| SMOX | Spermine oxidase |
| SOD | Superoxide Dismutase |
| SPSS | Statistical Package for the Social Sciences |
| STRING | Search Tool for the Retrieval of Interacting Genes/Proteins |
| Swissprot | Curated protein sequence database |
| TP53I3 | Tumor protein p53 inducible protein 3 |
| TRINITY_DNXXXX_cX_gX | Transcript identifier from Trinity assembly |
| UHPLC-MS | Ultra-High-Performance Liquid Chromatography-Mass Spectrometry |
| ULK1 | Unc-51 Like Autophagy Activating Kinase 1 |
| ULK2 | Unc-51 Like Autophagy Activating Kinase 2 |
| UQCR10 | Ubiquinol-Cytochrome C Reductase Complex III Subunit X |
| UQCRFS1 | Ubiquinol-Cytochrome C Reductase Rieske Iron-Sulfur Polypeptide 1 |
| VIP | Variable Importance in Projection |
| XCMS | Various forms (e.g., XCMS Online), platform for metabolomics data analysis |

## Supplemental results

### *S1.1. Functional annotation of assembled unigenes.*

In our study, a total of 39219 unigenes were identified in muscle tissues of hybrids after transcriptome assembly. For further functional prediction and classification, the 39219 unigenes were comparedwith BLASTx protein database and BLASTn protein nucleotide database. BLAST results showed that 23195, 17464, 13735, 15519, 21447,

and 18502 unigenes were annotated sequence matches with the NR, GO, KEGG, Pfam, eggNOG, and Swissprot databases, respectively (Figure S1 and Table S6).

*S1.2. GO, KEGG, eggNOG, NR functional classification.*

GO classification is a standardized system for classifying genes. Based on the functional annotations, 17464 unigenes were categorized into three main classes: biological processe, cellular component and molecular function. In addition, these unigenes were categorized into 55 subclasses. As shown in the Figure S2, 28, 3 and 24 subcategories were clustered in the biological process, cellular component and molecular function categories. First, in the category of biological processes, the most abundant were cellular processes, biological process, and molecular function, which contained 16863, 15708, and 13419 unigenes, respectively. Second, in the category of cellular components, 16863 unigenes were cellular anatomical entity, 6575 unigenes were categorized as protein-containing complex. Finally, in molecular functional , the binding sequences contained 13419 unigenes, and 6947 unigenes were predicted to have catalytic activity. These results indicate that most of the annotated unigenes are associated with various types of biological processes.

The KEGG pathway database is a collection of hand-drawn pathway maps representing of molecular interactions and response networks. According to the KO database, a total of 13735 single genes were categorized into 35 level 2 pathways, which were mainly categorized into Endocrine system (1298), Immune system (1441), and

Signal transduction (2615), as shown in Figure S3.

The unigenes were annotated with eggNOG comparisons, and the eggNOG numbers of the best comparison results were assigned to the corresponding unigenes. The correspondence between the eggNOG number and the eggNOG classification catalog was further utilized to categorize each unigenes to the eggNOG classification catalog, and the statistical results of the eggNOG classification annotations are shown in Figure S4.

By comparing the annotations with the NR, obtained the similarity between the unigene sequences of this species and those of its relatives, as well as the functional information of the genes of this species. The results of the comparison were counted and the distribution of species on the comparison, the E-value distribution of the comparison, and the distribution of sequence similarity were plotted (Figure S5).

**Table S1. Summary of annotation results.**

| Database | Number | Percentage |
|---|---|---|
| NR | 23195 | 59.14 |
| GO | 17464 | 44.53 |
| KEGG | 13735 | 35.02 |
| Pfam | 15519 | 39.57 |
| eggNOG | 21447 | 54.69 |
| Swissprot | 18502 | 47.18 |
| Inalldatabase | 9792 | 24.97 |

Database: the type of the database;Number: represents the number of Unigenes successfully annotated in the database;

Percentage (%): represents the proportion of the number of successfully annotated Unigenes in the database to the total number of Unigenes; In all database: the number of Unigenes that have been annotated in all databases.

**Table S2. Primers used in the quantitative PCR analysis.**

| Sequence ID | Gene name | Forward primers (5’-3’) | Reverse primers (5’-3’) | Amplicon size (bp) | Annealing Temperature (℃) |
|---|---|---|---|---|---|
| TRINITY_DN17724_c0_g1 | PRDX1 | CTGCCACCAGAGGGACCTTCAT | TCAGTGATGCTGCCGAGGAGTT | 144 | 60 |
| TRINITY_DN3853_c0_g1 | CRYZL1 | GGAGGTCTTGGAGTGGACATCGT | AGACGCTGCCTTCAGGAAGAGAA | 195 | 60 |
| TRINITY_DN6718_c0_g1 | TP53I3 | ACTCCACTCGCTCCAGCATGAA | TCAAGCCGCAGCTCTTCCTGA | 111 | 60 |
| TRINITY_DN1901_c0_g1 | FRRS1 | GCCGTTCGCTGTCATCACTCAT | TGCCTGCCCGCTGTGGATAA | 112 | 60 |
| TRINITY_DN12203_c0_g1 | SLC25A17 | CGTGCTGTCCGAACCGTAGAAC | TCCCTGCTGCTGGTGCTCAAT | 196 | 60 |
| TRINITY_DN481_c1_g1 | SMOX | TGCTGTATCCGCCTGAACGCTA | TCTCCGCCACAGTCTCGTCATC | 102 | 60 |
| TRINITY_DN4288_c0_g1 | PPARA | TCTCACAGGCAAGACCAGCACT | ACCGAGGCGTACTGACAGAACA | 177 | 60 |
| TRINITY_DN8897_c0_g1 | CYP1A1 | CGGCGTCATGTCCAGCATTTCT | CGGCACGGACCTCAACAAGATG | 172 | 60 |

**Table S3. Significantly different metabolites.**

| Name | *P*-value | M/z | Rt(s) | Mode | Regulation |
|---|---|---|---|---|---|
| Pro-leu | 3.13113E-06 | 229.15537 | 359.385 | POS | down |
| Pipamperone | 1.95804E-06 | 376.22137 | 274.7695 | POS | down |
| Pheniramine | 4.47007E-11 | 241.1536 | 329.1275 | POS | up |
| PC(16:0/16:0) | 0.000154618 | 756.55153 | 37.785 | POS | down |
| Oxybutynin | 0.00459907 | 358.23242 | 274.985 | POS | up |
| Octanoylcarnitine | 0.00206328 | 288.21592 | 195.32 | POS | up |
| Niacinamide | 0.02032737 | 123.05485 | 62.57 | POS | down |
| N-alpha-acetyl-l-lysine | 7.18942E-08 | 189.12206 | 383.374 | POS | up |
| Maltose | 2.89984E-06 | 365.104 | 386.191 | POS | up |
| Lys-Trp-Lys | 2.5422E-07 | 231.16888 | 375.0945 | POS | up |
| L-propionylcarnitine | 0.000664312 | 218.13856 | 270.797 | POS | up |
| L-Pipecolic acid | 0.000189552 | 147.1114 | 545.474 | POS | up |
| Lpc 18:2 | 0.029442535 | 520.33928 | 180.367 | POS | down |
| Lpc 18:1 | 0.008042272 | 522.35485 | 182.937 | POS | down |
| L-hydroxyarginine | 1.57536E-06 | 116.06955 | 300.6125 | POS | up |
| Leucine | 5.14293E-09 | 132.10114 | 257.829 | POS | up |
| L-carnosine | 1.36446E-07 | 156.10007 | 400.716 | POS | down |
| L-carnitine | 8.73908E-10 | 162.11247 | 344.503 | POS | down |
| Lauroyl-l-carnitine | 0.011338708 | 344.27761 | 175.234 | POS | up |
| L-Alanine | 6.70587E-08 | 134.0173 | 345.5455 | POS | up |
| Hexanoyl-l-carnitine | 1.34377E-06 | 260.18534 | 215.296 | POS | up |
| Glycerophosphocholine | 0.000883009 | 258.11047 | 378.834 | POS | up |

| | | | | | |
|---|---|---|---|---|---|
| Creatinine | 0.007469315 | 114.06491 | 169.1545 | POS | down |
| Betaine | 1.21915E-09 | 118.08579 | 265.699 | POS | down |
| Anserine | 0.000692774 | 241.15338 | 294.898 | POS | up |
| Adenosine | 1.19439E-08 | 285.127 | 341.168 | POS | up |
| Acetylcarnitine | 0.019844219 | 204.12318 | 295.648 | POS | down |
| 5-aminovaleric acid betaine | 1.30202E-11 | 160.13232 | 377.295 | POS | down |
| 4-hydroxy-l-isoleucine | 1.44561E-06 | 102.09 | 344.449 | POS | down |
| 3-methylglutarylcarnitine | 0.000151606 | 290.15863 | 240.4845 | POS | up |
| 2-methylbutyryl-l-carnitine | 0.011264427 | 246.17007 | 231.111 | POS | up |
| 2-amino-1-phenylethanol | 0.001327221 | 120.08028 | 253.254 | POS | down |
| 1-palmitoyl-sn-glycero-3-phosphocholine | 5.48167E-05 | 496.33923 | 185.527 | POS | down |
| 1-methyl-l-histidine | 3.29868E-10 | 170.09097 | 359.618 | POS | up |
| 1-(1z-hexadecenyl)-sn-glycero-3-phosphocholine | 1.72922E-05 | 480.34277 | 176.3895 | POS | down |
| 1-(1z-octadecenyl)-sn-glycero-3-phosphocholine | 5.18865E-05 | 508.37319 | 186.442 | POS | down |
| 1,2-diamino-2-methylpropane | 2.53059E-05 | 72.07986 | 292.159 | POS | up |
| 1,2-didocosahexaenoyl-sn-glycero-3-phosphocholine | 3.71492E-05 | 878.56648 | 36.9075 | POS | up |
| 1,2-dilinoleoyl-sn-glycero-3-phosphoethanolamine | 0.009183147 | 740.52001 | 141.9045 | POS | down |
| 1,5-pentanediamine | 0.000270682 | 86.09605 | 257.4895 | POS | up |
| Glycine | 1.22228E-11 | 74.025 | 366.992 | NEG | up |
| Uric acid | 5.31547E-08 | 167.04289 | 376.4485 | NEG | up |
| Thymol-beta-d-glucoside | 0.014334543 | 311.16801 | 28.044 | NEG | down |
| Thymidine | 0.000785912 | 241.08108 | 100.543 | NEG | down |
| Tartronate | 6.73181E-05 | 119.01609 | 357.92 | NEG | up |
| Rauwolscine | 0.005126182 | 353.19824 | 25.943 | NEG | down |

| | | | | | |
|---|---|---|---|---|---|
| Pseudouridine | 0.032967745 | 243.06109 | 157.704 | NEG | down |
| Proline | 0.000189202 | 114.05591 | 300.797 | NEG | up |
| Pi 38:5 | 0.001843076 | 883.53131 | 188.561 | NEG | down |
| Pi 38:4 | 0.034766111 | 885.54771 | 189.41 | NEG | down |
| Phosphoenolpyruvate | 0.020110465 | 166.97384 | 385.394 | NEG | up |
| Phosphatidylcholine lyso alkyl 16:0 | 6.61621E-06 | 540.36363 | 196.508 | NEG | down |
| Phenol | 0.031551264 | 93.04522 | 389.601 | NEG | down |
| Pg 36:3 | 0.018270137 | 771.5138 | 35.114 | NEG | down |
| Pc(18:1e/20-hdohe) | 0.00109881 | 892.60142 | 132.984 | NEG | up |
| Pc(16:1e/17-hdohe) | 0.001350039 | 864.57426 | 134.314 | NEG | up |
| Pc(16:0e/8-hepe) | 0.009678832 | 840.57383 | 137.199 | NEG | down |
| Pc 44:12 | 2.33367E-07 | 936.57157 | 129.0255 | NEG | up |
| Pc(18:1e/12-hete) | 0.002865656 | 868.60135 | 135.309 | NEG | up |
| Pc 40:7 | 0.000902646 | 890.58893 | 132.349 | NEG | up |
| Pc 34:3 | 0.015937339 | 814.55505 | 139.5975 | NEG | down |
| Pantetheine | 0.000190682 | 277.12069 | 47.915 | NEG | down |
| Ostruthin | 0.006438725 | 297.15137 | 28.535 | NEG | down |
| Nocardamine | 0.000251263 | 599.31697 | 242.286 | NEG | down |
| N-acetylhistidine | 0.000387905 | 196.0714 | 300.865 | NEG | up |
| Linoleic acid | 0.008028734 | 279.23255 | 42.686 | NEG | down |
| Ile-Pro | 2.20502E-08 | 227.06605 | 112.522 | NEG | down |
| Histamine | 0.010635142 | 110.07146 | 390.352 | NEG | down |
| Glutamine | 9.93079E-05 | 145.06148 | 376.005 | NEG | up |
| Glucoiberin | 0.008068552 | 358.01053 | 466.49 | NEG | up |

| | | | | | |
|---|---|---|---|---|---|
| Gamma-Glu-Cys | 1.12524E-05 | 249.04536 | 364.448 | NEG | down |
| Fahfa 38:4 | 0.026182916 | 585.48434 | 36.772 | NEG | down |
| Fahfa 36:3 | 0.026351461 | 559.46944 | 36.919 | NEG | down |
| Fahfa 36:2 | 0.017004919 | 561.48549 | 36.826 | NEG | down |
| Fa 18:2+2o | 0.009187685 | 311.2207 | 102.9365 | NEG | up |
| Erucic acid | 0.015221651 | 337.30909 | 36.143 | NEG | down |
| Eplerenone hydroxy acid | 0.021136675 | 337.20332 | 25.564 | NEG | down |
| Eicosenoic acid | 0.013354083 | 309.27778 | 37.623 | NEG | down |
| Cochlioquinone a | 2.00247E-07 | 531.29656 | 116.8585 | NEG | down |
| Cholesteryl sulfate | 0.032734831 | 465.30193 | 25.939 | NEG | down |
| Adynerin | 0.000107833 | 515.30029 | 88.558 | NEG | down |
| Acetoacetic acid | 0.00682839 | 101.02385 | 453.407 | NEG | down |
| Arachidonic Acid (peroxide free) | 3.09058E-05 | 303.23186 | 38.103 | NEG | down |
| Azelaic acid | 0.004831753 | 187.09651 | 336.876 | NEG | up |
| Benzenethiol, 4,4'-thiobis- | 1.96366E-05 | 248.99668 | 358.968 | NEG | down |
| Blood group b trisaccharide | 0.022739831 | 487.17435 | 402.232 | NEG | up |
| Cis,cis-muconic acid | 2.77815E-07 | 141.01689 | 356.105 | NEG | up |
| Cis-4,7,10,13,16,19-docosahexaenoic acid | 0.001877953 | 327.23161 | 37.957 | NEG | down |
| Lumichrome | 0.002874781 | 241.07161 | 62.9165 | NEG | down |
| Mestranol | 0.01103076 | 309.17266 | 25.939 | NEG | down |
| Mitragynine | 0.008609165 | 397.22345 | 27.189 | NEG | down |
| Nname,cis-9,10-Epoxystearic acid | 0.025905919 | 297.24184 | 36.36 | NEG | up |
| Valine | 2.78942E-05 | 116.07118 | 293.697 | NEG | up |

**Table S4. Amino acids, peptides, and analogue metabolites.**

| Name | *P*-value | M/z | Rt(s) | Mode | Regulation |
|---|---|---|---|---|---|
| 1-methyl-l-histidine | 3.29868E-10 | 170.09097 | 359.618 | POS | up |
| Betaine | 1.21915E-09 | 118.08579 | 265.699 | POS | down |
| Leucine | 5.14293E-09 | 132.10114 | 257.829 | POS | up |
| L-Alanine | 6.70587E-08 | 134.0173 | 345.5455 | POS | up |
| N-alpha-acetyl-l-lysine | 7.18942E-08 | 189.12206 | 383.374 | POS | up |
| Lys-Trp-Lys | 2.5422E-07 | 231.16888 | 375.0945 | POS | up |
| 4-hydroxy-l-isoleucine | 1.44561E-06 | 102.09 | 344.449 | POS | down |
| L-hydroxyarginine | 1.57536E-06 | 116.06955 | 300.6125 | POS | up |
| Pro-leu | 3.13113E-06 | 229.15537 | 359.385 | POS | down |
| Biocytin | 9.92345E-06 | 373.20707 | 384.187 | POS | down |
| 1-Methylhistidine | 0.000143951 | 170.09094 | 449.382 | POS | up |
| L-Pipecolic acid | 0.000189552 | 147.1114 | 545.474 | POS | up |
| Creatinine | 0.007469315 | 114.06491 | 169.1545 | POS | down |
| Glycine | 1.22228E-11 | 74.025 | 366.992 | NEG | up |
| Gamma-Glu-Cys | 1.12524E-05 | 249.04536 | 364.448 | NEG | down |
| Valine | 2.78942E-05 | 116.07118 | 293.697 | NEG | up |
| Glutamine | 9.93079E-05 | 145.06148 | 376.005 | NEG | up |
| Proline | 0.000189202 | 114.05591 | 300.797 | NEG | up |
| Pantetheine | 0.000190682 | 277.12069 | 47.915 | NEG | down |
| N-acetylhistidine | 0.000387905 | 196.0714 | 300.865 | NEG | up |

**Table S5. Fatty acid and derivatives metabolites.**

| Name | *P*-value | M/z | Rt(s) | Mode | Regulation |
|---|---|---|---|---|---|
| 5-aminovaleric acid betaine | 1.30202E-11 | 160.13232 | 377.295 | POS | down |
| Hexanoyl-l-carnitine | 1.34377E-06 | 260.18534 | 215.296 | POS | up |
| 4-chlorobutyric acid ethyl ester | 1.93735E-06 | 151.04635 | 363.316 | POS | up |
| Maltose | 2.89984E-06 | 365.104 | 386.191 | POS | up |
| 3-methylglutarylcarnitine | 0.000151606 | 290.15863 | 240.4845 | POS | up |
| 4-[5-[[4-[5-[acetyl(hydroxy)amino]pentylamino]-4-oxobutanoyl]-hydroxyamino]pentylamino]-4-oxobutanoic acid | 0.000419335 | 478.29111 | 190.557 | POS | down |
| L-propionylcarnitine | 0.000664312 | 218.13856 | 270.797 | POS | up |
| Octanoylcarnitine | 0.00206328 | 288.21592 | 195.32 | POS | up |
| Decanoyl-l-carnitine | 0.00300946 | 316.24653 | 182.974 | POS | up |
| 2-methylbutyryl-l-carnitine | 0.011264427 | 246.17007 | 231.111 | POS | up |
| Lauroyl-l-carnitine | 0.011338708 | 344.27761 | 175.234 | POS | up |
| (r)-butyrylcarnitine | 0.018614728 | 232.15417 | 248.953 | POS | up |
| Acetylcarnitine | 0.019844219 | 204.12318 | 295.648 | POS | down |
| Cis,cis-muconic acid | 2.77815E-07 | 141.01689 | 356.105 | NEG | up |
| Arachidonic Acid (peroxide free) | 3.09058E-05 | 303.23186 | 38.103 | NEG | down |
| (z)-5,8,11-trihydroxyoctadec-9-enoic acid | 6.44616E-05 | 329.23088 | 154.7525 | NEG | up |
| 9-(2,3-dihydroxypropoxy)-9-oxononanoic acid | 0.0002899 | 261.13266 | 183.844 | NEG | up |
| 2-methyl-3-hydroxybutyric acid | 0.001469228 | 117.05474 | 146.91 | NEG | up |

| | | | | | |
|---|---|---|---|---|---|
| Cis-4,7,10,13,16,19-docosahexaenoic acid | 0.001877953 | 327.23161 | 37.957 | NEG | down |
| Azelaic acid | 0.004831753 | 187.09651 | 336.876 | NEG | up |
| Eicosenoic acid | 0.013354083 | 309.27778 | 37.623 | NEG | down |
| Erucic acid | 0.015221651 | 337.30909 | 36.143 | NEG | down |
| Fahfa 36:2 | 0.017004919 | 561.48549 | 36.826 | NEG | down |
| Fahfa 38:4 | 0.026182916 | 585.48434 | 36.772 | NEG | down |
| Fahfa 36:3 | 0.026351461 | 559.46944 | 36.919 | NEG | down |
| 5-heptenoic acid, 7-[(1r,2r,3s,5s)-2-[(1e,3s)-3-(2,3-dihydro-1h-inden-2-yl)-3-hydroxy-1-propen-1-yl]-3-fluoro-5-hydroxycyclopentyl]-, (5z)- | 0.03061752 | 381.22886 | 25.943 | NEG | down |

**Table S6. Statistical assessment of sequencing data.**

| Sample | ReadsNo. | Bases(bp) | Q30(bp) | N(%) | Q20(%) | Q30(%) |
|---|---|---|---|---|---|---|
| Control_1 | 44071286 | 6610692900 | 6251966439 | 0.000206 | 98.19 | 94.57 |
| Control_2 | 41056226 | 6158433900 | 5840901874 | 0.003007 | 98.29 | 94.84 |
| Control_3 | 40682660 | 6102399000 | 5746313482 | 0.003028 | 97.99 | 94.16 |
| SA_1 | 42201462 | 6330219300 | 5981754731 | 0.003101 | 98.12 | 94.49 |
| SA_2 | 50002386 | 7500357900 | 7048874827 | 0.003065 | 97.9 | 93.98 |
| SA_3 | 48405422 | 7260813300 | 6826212322 | 0.003123 | 97.93 | 94.01 |

Read number: Total number of pair end reads in Clean Data;
Base Number: total number of bases in Clean Data;
N (%): Percentage of fuzzy bases;
Q20(%): The percentage of bases with quality value≥20;
Q30(%): The percentage of bases with quality value≥30.

**Table S7. Overall sequence statistics.**

| Contig | Transcript | Unigene |
|---|---|---|
| TotalLength(bp) | 130804041 | 48425964 |
| SequenceNumber | 84462 | 39219 |
| Max.Length(bp) | 36555 | 36555 |
| MeanLength(bp) | 1548.67 | 1234.76 |
| N50(bp) | 2517 | 2087 |
| N50SequenceNo. | 15374 | 6603 |
| N90(bp) | 632 | 469 |
| N90SequenceNo. | 54043 | 26187 |
| GC% | 45.04 | 44.82 |

Total Length (bp): total length of the sequence;
Sequence Number: total number of sequences;
Max. Length (bp): maximum length of the sequence;
Mean Length: average length of the sequence;
N50 (bp): arrange all sequences from longest to shortest, add the lengths of the sequences in that order, and when the added length reaches 50% of the total length of the sequence, the length of the last sequence;
N90 (bp): Arrange all sequences from longest to shortest, add the lengths of the sequences in that order, and when the summed length reaches 90% of the total length of the sequence, the length of the last sequence;
N50 Sequence No.: the total number of sequences with length greater than N50;

N90 Sequence No.: the total number of sequences whose length is greater than N90;
GC%: the GC content of the sequence.

**Table S8. Representative oxidation-reduction-related DEGs.**

| Gene ID | Description | Gene name | log2 Fold Change | Padj | Regulation |
|---|---|---|---|---|---|
| TRINITY_DN10307_c0_g1 | NAD(P)H dehydrogenase [quinone] 1 | NQO1 | 1.459013394 | 1.44E-01 | up |
| TRINITY_DN10936_c0_g1 | Pyruvate dehydrogenase E1 component subunit alpha | PDHA1 | 3.310360542 | 0.252100481 | up |
| TRINITY_DN11424_c0_g1 | Autophagy-related protein 2 homolog A | ATG2A | 1.015800141 | 0.001068409 | up |
| TRINITY_DN12203_c0_g1 | Peroxisomal membrane protein PMP34 | SLC25A17 | 1.481068841 | 0.168897974 | up |
| TRINITY_DN13109_c0_g1 | SH3 and PX domain-containing protein 2A | SH3PXD2A | 1.03527703 | 0.210507011 | up |
| TRINITY_DN1701_c0_g1 | Autophagy-related protein 9A | ATG9A | 1.49778288 | 1.2785E-10 | up |
| TRINITY_DN17724_c0_g1 | Peroxiredoxin-1 | PRDX1 | 1.689901429 | 0.122039617 | up |
| TRINITY_DN1946_c0_g1 | Insulin receptor substrate 2 | IRS2 | 1.222588373 | 1.71359E-08 | up |
| TRINITY_DN337_c5_g1 | nuclear receptor subfamily 1 group D member 1 | NR1D1 | 2.283043178 | 5.79533E-08 | up |
| TRINITY_DN3853_c0_g1 | Quinone oxidoreductase-like protein 1 | CRYZL1 | 1.024045186 | 0.364829888 | up |
| TRINITY_DN4116_c0_g1 | FLCN_HUMAN Folliculin | FLCN | 1.055735903 | 0.015142853 | up |
| TRINITY_DN4649_c0_g1 | Cellular tumor antigen p53 | TP53 | 1.804785652 | 0.03286117 | up |
| TRINITY_DN481_c1_g1 | Spermine oxidase | SMOX | 1.094510264 | 1.09394E-07 | up |
| TRINITY_DN4932_c0_g2 | LYR motif-containing protein 5A | LYRM5A | 1.012311839 | 0.00576702 | up |
| TRINITY_DN512_c0_g1 | Serine/threonine-protein kinase ULK2 | ULK2 | 1.47394921 | 1.06579E-13 | up |
| TRINITY_DN519_c1_g1 | Forkhead box protein O3 | FOXO3 | 1.611168619 | 3.3171E-07 | up |
| TRINITY_DN5225_c0_g1 | Ferredoxin-2, mitochondrial | FDX2 | 1.387380654 | 0.044825292 | up |
| TRINITY_DN5705_c0_g1 | Cystathionine beta-synthase | CBS | 1.186487704 | 0.009730354 | up |
| TRINITY_DN5903_c0_g1 | Mycocerosic acid synthase-like polyketide synthase | PKS5 | 2.056041347 | 7.00151E-13 | up |

| | | | | | |
|---|---|---|---|---|---|
| TRINITY_DN6023_c0_g1 | Cytosolic 10-formyltetrahydrofolate dehydrogenase | ALDH1L1 | 1.300084654 | 0.329243075 | up |
| TRINITY_DN6116_c0_g1 | Glutathione reductase | GSR | 1.280724934 | 0.038709941 | up |
| TRINITY_DN6718_c0_g1 | Quinone oxidoreductase PIG3 | TP53I3 | 1.648356655 | 0.22252775 | up |
| TRINITY_DN7530_c1_g1 | Autophagy-related protein 2 homolog B | ATG2B | 1.384410189 | 4.765E-07 | up |
| TRINITY_DN8517_c0_g1 | Carnitine O-palmitoyltransferase 1 | CPT1A | 1.177715381 | 0.18163243 | up |
| TRINITY_DN8897_c0_g1 | Cytochrome P450 1A1 | CYP1A1 | -2.889166214 | 1.51911E-05 | down |
| TRINITY_DN4288_c0_g1 | Peroxisome proliferator-activated receptor alpha | PPARA | -1.599020467 | 0.002916593 | down |
| TRINITY_DN10173_c0_g1 | ERO1-like protein alpha | ERO1A | -1.089379236 | 0.069153308 | down |
| TRINITY_DN1037_c0_g1 | Glycerol-3-phosphate dehydrogenase [NAD(+)] | GPD1 | -1.133245989 | 7.01131E-18 | down |
| TRINITY_DN108_c1_g1 | EGLN1_MOUSE Egl nine homolog 1 | EGLN1 | -1.113447178 | 2.43607E-10 | down |
| TRINITY_DN11157_c0_g1 | Short-chain dehydrogenase/reductase 3 | DHRS3 | -1.076899282 | 0.115449696 | down |
| TRINITY_DN11238_c0_g1 | Arginine-hydroxylase NDUFAF5, mitochondrial | NDUFAF5 | -1.158591451 | 1.30231E-05 | down |
| TRINITY_DN11368_c0_g1 | Glyceraldehyde-3-phosphate dehydrogenase | GAPDH | -4.06038817 | 0.038387765 | down |
| TRINITY_DN12884_c0_g1 | Fatty acyl-CoA reductase 1 | FAR1 | -1.604688267 | 0.064416263 | down |
| TRINITY_DN12912_c0_g1 | SH3 domain-binding glutamic acid-rich-like protein 3 | SH3BGRL3 | -2.571658734 | 0.001194374 | down |
| TRINITY_DN13486_c0_g1 | L-lactate dehydrogenase B-B chain | LDHBB | -4.311023397 | 0.376563587 | down |
| TRINITY_DN1361_c0_g1 | Adenylosuccinate lyase | ADSL | -1.015417942 | 4.94042E-09 | down |
| TRINITY_DN1467_c0_g1 | Prolyl hydroxylase | EGLN2 | -1.706531808 | 6.79924E-17 | down |
| TRINITY_DN148_c2_g1 | Phosphorylase b kinase regulatory subunit beta | PHKB | -1.077059108 | 3.24371E-14 | down |
| TRINITY_DN15212_c0_g1 | Alpha-ketoglutarate-dependent dioxygenase alkB homolog 4 | ALKBH4 | -1.482005285 | 0.13579846 | down |
| TRINITY_DN15988_c0_g1 | Beta-enolase | ENO3 | -1.841567505 | 0.01727297 | down |
| TRINITY_DN1687_c2_g1 | HIG1 domain family member 1A, mitochondrial | HIGD1A | -1.199299815 | 6.96853E-11 | down |
| TRINITY_DN18882_c1_g1 | RAC-alpha serine/threonine-protein kinase | AKT1 | -1.135963714 | 0.026267496 | down |

| | | | | | |
|---|---|---|---|---|---|
| TRINITY_DN1892_c0_g2 | Mitogen-activated protein kinase 14A | MAPK14A | -1.201491482 | 0.00307253 | down |
| TRINITY_DN1901_c0_g1 | Putative ferric-chelate reductase 1 | FRRS1 | -1.261053063 | 0.000150502 | down |
| TRINITY_DN1955_c0_g1 | Very long-chain acyl-CoA synthetase | SLC27A2 | -2.189144693 | 4.19507E-09 | down |
| TRINITY_DN1986_c0_g1 | Glycogen phosphorylase | PYGM | -1.021774084 | 1.12394E-14 | down |
| TRINITY_DN201_c0_g1 | NADH dehydrogenase [ubiquinone] 1 alpha subcomplex subunit 6 | NDUFA6 | -1.051797467 | 0.02710401 | down |
| TRINITY_DN20578_c0_g1 | Lanosterol 14-alpha demethylase | CYP51 | -4.676157727 | 0.192534081 | down |
| TRINITY_DN20752_c0_g1 | Glutathione S-transferase P | GSTP1 | -5.376127922 | 0.226546414 | down |
| TRINITY_DN2080_c0_g1 | Fructose-bisphosphate aldolase B | ALDOB | -1.54917801 | 0.000112724 | down |
| TRINITY_DN20851_c0_g2 | Sarcoplasmic/endoplasmic reticulum calcium ATPase 2 | ATP2A2 | -1.249607628 | 0.022356499 | down |
| TRINITY_DN20904_c0_g2 | Isocitrate dehydrogenase [NAD] subunit gamma | IDH3G | -1.682637415 | 0.295316232 | down |
| TRINITY_DN2168_c0_g2 | Hexokinase-1 | HK1 | -1.807760245 | 1.21905E-24 | down |
| TRINITY_DN2310_c0_g1 | Cysteine dioxygenase type 1 | CDO1 | -1.004227642 | 0.000971655 | down |
| TRINITY_DN23517_c0_g2 | NADH dehydrogenase [ubiquinone] 1 alpha subcomplex subunit 11 | NDUFA11 | -1.035267154 | 0.004210745 | down |
| TRINITY_DN23547_c0_g1 | Hexokinase-4 | GCK | -7.800791323 | 6.98105E-05 | down |
| TRINITY_DN23968_c0_g1 | Sarcoplasmic/endoplasmic reticulum calcium ATPase 1 | ATP2A1 | -1.597847414 | 2.62509E-24 | down |
| TRINITY_DN2488_c0_g1 | Mitochondrial dicarboxylate carrier | SLC25A10 | -1.167538757 | 0.093036858 | down |
| TRINITY_DN2517_c0_g1 | Cytochrome b-c1 complex subunit 9 | UQCR10 | -1.183617329 | 0.002700681 | down |
| TRINITY_DN2587_c0_g1 | Period circadian protein homolog 2 | PER2 | -1.69653865 | 5.79976E-18 | down |
| TRINITY_DN26095_c0_g1 | Apolipoprotein A-I | APOA1 | -3.738300144 | 0.014449992 | down |
| TRINITY_DN2616_c0_g1 | Cytochrome c oxidase subunit 7B | COX7B | -1.051851261 | 0.009762608 | down |

| | | | | | |
|---|---|---|---|---|---|
| TRINITY_DN2639_c0_g1 | E3 ubiquitin-protein ligase RNF182 | RNF182 | -1.570044284 | 0.007643147 | down |
| TRINITY_DN26685_c0_g1 | 4-hydroxyphenylpyruvate dioxygenase | HPD | -2.320316146 | 0.000336883 | down |
| TRINITY_DN2708_c2_g1 | NADH dehydrogenase [ubiquinone] 1 beta subcomplex subunit 3 | NDUFB3 | -1.140637455 | 0.000410201 | down |
| TRINITY_DN28164_c0_g1 | Mitochondrial intermembrane space import and assembly protein 40 | CHCHD4 | -1.998628953 | 1.56839E-05 | down |
| TRINITY_DN2823_c0_g1 | Lysine-specific demethylase 6B | KDM6B | -1.609651131 | 6.98105E-05 | down |
| TRINITY_DN2878_c0_g1 | Lysyl oxidase homolog 4 | LOXL4 | -2.078640552 | 0.211459967 | down |
| TRINITY_DN2958_c0_g1 | Insulin receptor substrate 1-B | IRS1-B | -2.435894939 | 1.80999E-24 | down |
| TRINITY_DN30025_c0_g1 | Peroxiredoxin-like 2C | PRXL2C | -1.401255943 | 0.320456681 | down |
| TRINITY_DN3110_c0_g1 | Acyl carrier protein | NDUFAB1 | -1.051150249 | 0.001123327 | down |
| TRINITY_DN3377_c0_g1 | L-threonine 3-dehydrogenase | TDH | -2.667470016 | 3.87316E-17 | down |
| TRINITY_DN34085_c0_g1 | Ceruloplasmin | CP | -1.533101192 | 0.314309152 | down |
| TRINITY_DN34157_c0_g1 | Glucose-6-phosphatase catalytic subunit 1 | G6PC1 | -3.140517196 | 0.005555497 | down |
| TRINITY_DN3459_c3_g1 | Glucose 1,6-bisphosphate synthase | PGM2L1 | -1.168773394 | 4.26542E-06 | down |
| TRINITY_DN34911_c0_g1 | Complex III assembly factor LYRM7 | LYRM7 | -1.344188044 | 0.000133121 | down |
| TRINITY_DN3518_c0_g1 | Maleylacetoacetate isomerase | GSTZ1 | -1.311311155 | 0.240213632 | down |
| TRINITY_DN3549_c0_g1 | Cytochrome c oxidase subunit 5A | COX5A | -1.182433074 | 0.005744537 | down |
| TRINITY_DN355_c0_g1 | NADH dehydrogenase [ubiquinone] 1 alpha subcomplex subunit 13 | NDUFA13 | -1.196527777 | 0.001195241 | down |
| TRINITY_DN3581_c0_g1 | Phosphoglycerate mutase 2 | PGAM2 | -1.117188618 | 3.62458E-14 | down |
| TRINITY_DN35_c0_g2 | Glycogen debranching enzyme | AGL | -1.262029671 | 2.18399E-18 | down |
| TRINITY_DN3655_c0_g1 | Sestrin-2 | SESN2 | -1.524951823 | 3.02286E-07 | down |
| TRINITY_DN3723_c0_g1 | Sphingolipid delta(4)-desaturase DES1 | DEGS1 | -1.172202404 | 0.000996089 | down |

| | | | | | |
|---|---|---|---|---|---|
| TRINITY_DN3728_c0_g1 | Adiponectin | ADIPOQ | -1.433167223 | 0.028461719 | down |
| TRINITY_DN3831_c0_g1 | Glucose-6-phosphate isomerase | GPI | -1.088224515 | 0.253689467 | down |
| TRINITY_DN3845_c0_g1 | Cytochrome b-c1 complex subunit Rieske | UQCRFS1 | -1.021930015 | 0.001226754 | down |
| TRINITY_DN408_c0_g1 | Coenzyme Q-binding protein COQ10 homolog B | COQ10B | -2.699217269 | 1.0246E-83 | down |
| TRINITY_DN4122_c0_g1 | L-lactate dehydrogenase B-A chain | LDHBA | -1.143927963 | 0.002396212 | down |
| TRINITY_DN42159_c0_g1 | NADH dehydrogenase [ubiquinone] 1 beta subcomplex subunit 11 | NDUFB11 | -1.008402376 | 4.81135E-08 | down |
| TRINITY_DN42587_c0_g1 | Methylsterol monooxygenase 1 | MSMO1 | -4.083773583 | 0.366208065 | down |
| TRINITY_DN4519_c0_g1 | Glutathione S-transferase kappa 1 | GSTK1 | -4.234650678 | 0.310696341 | down |
| TRINITY_DN46314_c0_g1 | Microsomal glutathione S-transferase 3 | MGST3 | -2.122774357 | 0.388869354 | down |
| TRINITY_DN4746_c0_g1 | L-2-hydroxyglutarate dehydrogenase | L2HGDH | -1.038023076 | 0.099844023 | down |
| TRINITY_DN4756_c0_g1 | 4-hydroxyphenylpyruvate dioxygenase-like protein | HPDL | -1.595223226 | 0.017909694 | down |
| TRINITY_DN476_c0_g1 | NADH dehydrogenase [ubiquinone] 1 beta subcomplex subunit 4 | NDUFB4 | -1.050903904 | 3.24386E-06 | down |
| TRINITY_DN4927_c0_g1 | Nuclear receptor subfamily 4 group A member 3 | NR4A3 | -2.995077104 | 6.86475E-33 | down |
| TRINITY_DN519_c2_g1 | Protein phosphatase 1 regulatory subunit 3A | PPP1R3A | -1.058083569 | 2.34826E-05 | down |
| TRINITY_DN5277_c0_g1 | NADH dehydrogenase [ubiquinone] 1 beta subcomplex subunit 9 | NDUFB9 | -1.284573064 | 0.006167326 | down |
| TRINITY_DN5607_c0_g1 | Pyruvate kinase PKM | PKM | -1.585753108 | 0.159708993 | down |
| TRINITY_DN5816_c0_g1 | Cytochrome c1 | CYC1 | -1.030249639 | 7.14398E-07 | down |
| TRINITY_DN5884_c0_g1 | UDP-glucose 6-dehydrogenase | UGDH | -1.932164119 | 0.006875334 | down |
| TRINITY_DN609_c2_g1 | Proline dehydrogenase 1 | PRODH | -4.885593514 | 3.64483E-17 | down |
| TRINITY_DN6109_c0_g1 | Acyl-CoA 6-desaturase | FADS2 | -2.014639318 | 4.43528E-06 | down |
| TRINITY_DN6209_c0_g1 | 1,25-dihydroxyvitamin D(3) 24-hydroxylase | CYP24A1 | -2.221179799 | 0.018187523 | down |

| | | | | | |
|---|---|---|---|---|---|
| TRINITY_DN6459_c0_g1 | Evolutionarily conserved signaling intermediate in Toll pathway | ECSIT | -1.106986805 | 0.002681359 | down |
| TRINITY_DN6804_c0_g1 | ERO1-like protein beta | ERO1B | -1.261564887 | 0.005269226 | down |
| TRINITY_DN6977_c0_g1 | Ferritin heavy chain | FTH1 | -3.966733018 | 0.011568155 | down |
| TRINITY_DN7036_c0_g1 | Lysine-specific demethylase 9 | RSBN1 | -1.415486146 | 0.000318184 | down |
| TRINITY_DN711_c0_g1 | NADH-ubiquinone oxidoreductase chain 5 | MT-ND5 | -1.034303925 | 1.0473E-11 | down |
| TRINITY_DN7460_c0_g1 | Alpha-enolase | ENO1 | -1.109955613 | 3.52366E-17 | down |
| TRINITY_DN7736_c0_g1 | AMP-activated protein kinase subunit gamma-2 | PRKAG2 | -1.129015964 | 0.201391519 | down |
| TRINITY_DN7763_c0_g1 | Cytochrome c oxidase subunit 8B | COX8B | -1.001712876 | 7.55397E-05 | down |
| TRINITY_DN796_c3_g1 | Bifunctional methylenetetrahydrofolate dehydrogenase/cyclohydrolase | MTHFD2 | -1.104014282 | 0.00648088 | down |
| TRINITY_DN79_c0_g1 | ATP-dependent 6-phosphofructokinase | PFKM | -1.288877148 | 1.17471E-22 | down |
| TRINITY_DN8027_c0_g2 | mismatch-specific thymine DNA glycosylase | TDG | -1.396605166 | 0.001371246 | down |
| TRINITY_DN8035_c1_g1 | protein C1494.01 | SPCC1494.01 | -5.136380409 | 3.48313E-20 | down |
| TRINITY_DN8273_c0_g1 | Vitamin D 25-hydroxylase | CYP2R1 | -1.79280713 | 0.002823948 | down |
| TRINITY_DN894_c0_g1 | UTP--glucose-1-phosphate uridylyltransferase | UGP2 | -1.226141349 | 1.04654E-15 | down |
| TRINITY_DN8993_c0_g1 | Methanethiol oxidase | SELENBP1 | -1.162902685 | 0.016786634 | down |
| TRINITY_DN9020_c1_g1 | Cytochrome c oxidase subunit 2 | MT-CO2 | -1.078093388 | 7.56741E-10 | down |
| TRINITY_DN926_c0_g2 | RAC-beta serine/threonine-protein kinase | AKT2 | -1.330040334 | 7.05646E-10 | down |
| TRINITY_DN985_c0_g1 | Cytochrome c oxidase subunit 7C | COX7C | -1.221053318 | 0.009961293 | down |
| TRINITY_DN9921_c0_g1 | Laforin | EPM2A | -1.218095411 | 0.176209482 | down |
| TRINITY_DN9998_c0_g1 | Carbonyl reductase family member 4 | CBR4 | -1.104422096 | 0.175064099 | down |

15

**Table S9. Representative ion transport-related DEGs.**

| Gene ID | Description | Gene name | log2 Fold Change | Padj | Regulation |
|---|---|---|---|---|---|
| TRINITY_DN228_c0_g1 | Protein-lysine methyltransferase METTL21C | METTL21C | 2.23003088 | 4.12495E-22 | up |
| TRINITY_DN1155_c1_g1 | Sodium/calcium exchanger 3 | SLC8A3 | 1.333187158 | 3.37077E-13 | up |
| TRINITY_DN3727_c0_g1 | Mid1-interacting protein 1-B | MID1IP1B | 1.035865467 | 4.6249E-13 | up |
| TRINITY_DN66_c0_g1 | Voltage-dependent calcium channel gamma-1 subunit | CACNG1 | 1.037085203 | 1.10989E-11 | up |
| TRINITY_DN542_c4_g1 | Kelch-like protein 24 | KLHL24 | 1.158336724 | 2.30402E-11 | up |
| TRINITY_DN2776_c0_g2 | E3 ubiquitin-protein ligase NEDD4 | NEDD4 | 1.724895267 | 1.34725E-09 | up |
| TRINITY_DN1946_c0_g1 | Insulin receptor substrate 2 | IRS2 | 1.222588373 | 1.71359E-08 | up |
| TRINITY_DN1893_c0_g1 | A-kinase anchor protein 6 | AKAP6 | 2.290345062 | 1.79362E-08 | up |
| TRINITY_DN81_c0_g2 | Voltage-dependent calcium channel gamma-6 subunit | CACNG6 | 1.024020523 | 8.01325E-08 | up |
| TRINITY_DN5362_c0_g1 | Solute carrier family 15 member 4 | SLC15A4 | 1.845555996 | 8.84996E-07 | up |
| TRINITY_DN148_c14_g1 | Voltage-dependent calcium channel gamma-3 subunit | CACNG3 | 1.260056251 | 1.30908E-05 | up |
| TRINITY_DN4260_c0_g2 | Voltage-dependent T-type calcium channel subunit alpha-1H | CACNA1H | 3.50225198 | 0.000163856 | up |
| TRINITY_DN13132_c0_g2 | Protein Shroom2 | SHROOM2 | 2.471854607 | 0.000499464 | up |
| TRINITY_DN1974_c0_g1 | Solute carrier family 13 member 1 | SLC13A1 | 1.27314896 | 0.00096702 | up |
| TRINITY_DN10690_c0_g1 | 3-phosphoinositide-dependent protein kinase 1 | PDPK1 | 2.291186344 | 0.001523241 | up |
| TRINITY_DN5035_c0_g1 | Reduced folate transporter | SLC19A1 | 1.14331765 | 0.001642048 | up |
| TRINITY_DN1499_c0_g1 | Large neutral amino acids transporter small subunit 4 | SLC43A2 | 2.009745096 | 0.002022022 | up |
| TRINITY_DN5330_c0_g1 | Myotubularin-related protein 6 | MTMR6 | 1.095970059 | 0.002891116 | up |
| TRINITY_DN9554_c0_g1 | Ryanodine receptor 3 | RYR3 | 5.080114691 | 0.005440681 | up |
| TRINITY_DN11394_c0_g2 | Chloride transport protein 6 | CLCN6 | 1.654326418 | 0.013900493 | up |

| | | | | | |
|---|---|---|---|---|---|
| TRINITY_DN9898_c0_g2 | Cell cycle control protein 50A | TMEM30A | 1.071044674 | 0.015704454 | up |
| TRINITY_DN4378_c0_g1 | Sodium- and chloride-dependent glycine transporter 1 | SLC6A9 | 1.234882858 | 0.020509215 | up |
| TRINITY_DN3574_c0_g1 | Sodium-dependent lysophosphatidylcholine symporter 1-B | MFSD2AB | -3.451535077 | 1.44277E-37 | down |
| TRINITY_DN1208_c0_g1 | Monocarboxylate transporter 1 | SLC16A1 | -2.451699824 | 5.56264E-34 | down |
| TRINITY_DN2588_c0_g1 | ATP-binding cassette sub-family C member 5 | ABCC5 | -1.765657561 | 1.93798E-27 | down |
| TRINITY_DN29974_c0_g1 | Solute carrier family 2, facilitated glucose transporter member 4 | SLC2A4 | -1.639304652 | 9.22058E-25 | down |
| TRINITY_DN2168_c0_g2 | Hexokinase-1 | HK1 | -1.807760245 | 1.21905E-24 | down |
| TRINITY_DN23968_c0_g1 | Sarcoplasmic/endoplasmic reticulum calcium ATPase 1 | ATP2A1 | -1.597847414 | 2.62509E-24 | down |
| TRINITY_DN156_c0_g2 | Sodium- and chloride-dependent creatine transporter 1 | SLC6A8 | -1.612389133 | 1.08746E-23 | down |
| TRINITY_DN2587_c0_g1 | Period circadian protein homolog 2 | PER2 | -1.69653865 | 5.79976E-18 | down |
| TRINITY_DN1856_c0_g1 | Two pore channel protein 2 | TPCN2 | -1.579065455 | 4.95133E-16 | down |
| TRINITY_DN492_c1_g1 | Mitochondrial glutamate carrier 1 | SLC25A22 | -1.534777673 | 7.38261E-15 | down |
| TRINITY_DN605_c0_g1 | Transferrin receptor protein 1 | TFRC | -1.315305286 | 2.0355E-13 | down |
| TRINITY_DN4406_c0_g1 | Calcium-activated potassium channel subunit beta-2 | KCNMB2 | -1.600423939 | 7.01142E-13 | down |
| TRINITY_DN926_c0_g2 | RAC-beta serine/threonine-protein kinase | AKT2 | -1.330040334 | 7.05646E-10 | down |
| TRINITY_DN371_c0_g2 | Phosphatidylinositol-binding clathrin assembly protein | PICALM | -1.141130148 | 7.22142E-10 | down |
| TRINITY_DN9020_c1_g1 | Cytochrome c oxidase subunit 2 | MT-CO2 | -1.078093388 | 7.56741E-10 | down |
| TRINITY_DN5684_c1_g1 | Caveolin-1 | CAV1 | -1.230172937 | 1.48747E-09 | down |
| TRINITY_DN1955_c0_g1 | Very long-chain acyl-CoA synthetase | SLC27A2 | -2.189144693 | 4.19507E-09 | down |
| TRINITY_DN747_c0_g1 | Solute carrier family 41 member 1 | SLC41A1 | -1.071319676 | 6.0284E-09 | down |

| | | | | | |
|---|---|---|---|---|---|
| TRINITY_DN974_c0_g1 | Filamin-A | FLNA | -1.095763309 | 1.19708E-08 | down |
| TRINITY_DN300_c0_g1 | Major facilitator superfamily domain-containing protein 10 | MFSD10 | -1.731398609 | 1.82646E-08 | down |
| TRINITY_DN1386_c1_g1 | Sodium/potassium-transporting ATPase subunit beta-1 | ATP1B1 | -1.054762349 | 3.60086E-08 | down |
| TRINITY_DN8492_c0_g1 | ATP synthase subunit d | ATP5PD | -1.013667699 | 4.96632E-08 | down |
| TRINITY_DN5816_c0_g1 | Cytochrome c1 | CYC1 | -1.030249639 | 7.14398E-07 | down |
| TRINITY_DN2751_c0_g1 | ATP synthase subunit O | ATP5PO | -1.155334401 | 6.32823E-06 | down |
| TRINITY_DN6180_c0_g1 | Na(+)/H(+) exchange regulatory cofactor NHE-RF1 | SLC9A3R1 | -1.299212364 | 7.72954E-06 | down |
| TRINITY_DN35630_c0_g1 | Mitochondrial uncoupling protein 2 | UCP2 | -4.034651735 | 7.75289E-06 | down |
| TRINITY_DN5167_c0_g1 | P2X purinoceptor 5 | P2RX5 | -1.05123388 | 1.27467E-05 | down |
| TRINITY_DN6721_c0_g1 | ATP synthase subunit gamma | ATP5F1C | -1.031840958 | 2.36196E-05 | down |
| TRINITY_DN8297_c0_g1 | Voltage-dependent anion-selective channel protein 2 | VDAC2 | -1.17286391 | 5.88398E-05 | down |
| TRINITY_DN7763_c0_g1 | Cytochrome c oxidase subunit 8B | COX8B | -1.001712876 | 7.55397E-05 | down |
| TRINITY_DN3194_c0_g1 | ATP synthase subunit beta | ATP5F1B | -1.121740474 | 7.71328E-05 | down |
| TRINITY_DN9992_c0_g1 | Inward rectifier potassium channel 2 | KCNJ2 | -2.014403405 | 0.000101571 | down |
| TRINITY_DN7912_c0_g1 | Integrin beta-3 | ITGB3 | -1.846765898 | 0.000221409 | down |
| TRINITY_DN1944_c0_g1 | Alpha-actinin-2 | ACTN2 | -1.33629175 | 0.000551658 | down |
| TRINITY_DN8597_c0_g1 | Retinol-binding protein 4-A | RBP4A | -2.41219275 | 0.000763518 | down |
| TRINITY_DN361_c0_g1 | ATP synthase subunit delta | ATP5F1D | -1.199982914 | 0.000801802 | down |
| TRINITY_DN8636_c0_g1 | Cationic amino acid transporter 3 | SLC7A3 | -3.249503303 | 0.000818996 | down |
| TRINITY_DN2749_c0_g1 | ATP synthase subunit f | ATP5MF | -1.352368304 | 0.001043342 | down |
| TRINITY_DN9264_c0_g1 | Cytochrome c oxidase subunit 5A | COX5A | -1.084459906 | 0.001098125 | down |
| TRINITY_DN16571_c0_g1 | Prothrombin | F2 | -5.778209981 | 0.001591226 | down |

| | | | | | |
|---|---|---|---|---|---|
| TRINITY_DN4171_c0_g1 | Natural resistance-associated macrophage protein 2 | SLC11A2 | -1.111208987 | 0.001700056 | down |
| TRINITY_DN6915_c2_g1 | ATP synthase F(0) complex subunit C2 | ATP5MC2 | -1.437093646 | 0.002581803 | down |
| TRINITY_DN545_c0_g1 | synthase-coupling factor 6 | ATP5PF | -1.013300435 | 0.002956074 | down |
| TRINITY_DN2616_c0_g1 | Cytochrome c oxidase subunit 7B | COX7B | -1.051851261 | 0.009762608 | down |
| TRINITY_DN985_c0_g1 | Cytochrome c oxidase subunit 7C | COX7C | -1.221053318 | 0.009961293 | down |
| TRINITY_DN1689_c3_g1 | Four and a half LIM domains protein 1 | FHL1 | -1.240735791 | 0.011117834 | down |
| TRINITY_DN13529_c0_g1 | Sodium channel subunit beta-3 | SCN3B | -1.783807099 | 0.016147912 | down |
| TRINITY_DN2654_c0_g1 | Sarcoplasmic/endoplasmic reticulum calcium ATPase 2 | ATP2A2 | -1.851374511 | 0.016289276 | down |
| TRINITY_DN6797_c0_g1 | Neutral amino acid transporter A | SLC1A4 | -1.119701671 | 0.019792755 | down |
| TRINITY_DN4936_c0_g2 | ADP/ATP translocase 2 | SLC25A5 | -1.667608006 | 0.021118919 | down |
| TRINITY_DN9760_c0_g2 | Transient receptor potential cation channel subfamily M member 4 | TRPM4 | -1.090057779 | 0.025442163 | down |
| TRINITY_DN18882_c1_g1 | RAC-alpha serine/threonine-protein kinase | AKT1 | -1.135963714 | 0.026267496 | down |
| TRINITY_DN3465_c0_g1 | Serotransferrin-1 | TF1 | -2.977000211 | 0.033439432 | down |
| TRINITY_DN26956_c0_g2 | Potassium voltage-gated channel subfamily A member 4 | KCNA4 | -2.351568275 | 0.034800022 | down |
| TRINITY_DN5434_c0_g1 | Calsequestrin-2 | CASQ2 | -1.655241897 | 0.042712675 | down |
| TRINITY_DN4130_c1_g1 | Anoctamin-8 | ANO8 | -1.013165522 | 0.042907784 | down |

**Table S10. Representative complement and coagulation cascades-related DEGs.**

| Gene ID | Description | Gene name | log2 Fold Change | Padj | Regulation |
|---|---|---|---|---|---|
| TRINITY_DN6608_c0_g1 | Vitamin K-dependent protein S | PROS1 | 1.020968908 | 0.270050036 | up |
| TRINITY_DN1198_c0_g1 | Complement C4-B | C4B | 1.287031478 | 1.20088E-13 | up |
| TRINITY_DN21483_c0_g1 | Complement component C6 | C6 | 1.624251356 | 0.303919032 | up |
| TRINITY_DN9841_c0_g1 | Complement factor I | CFI | -3.761177077 | 0.008478616 | down |
| TRINITY_DN27792_c0_g2 | Kininogen-2 | KNG2 | -2.778259485 | 0.000352966 | down |
| TRINITY_DN6049_c0_g1 | Urokinase-type plasminogen activator | PLAU | -1.663849916 | 0.037850962 | down |
| TRINITY_DN21208_c0_g1 | Plasminogen | PLG | -3.702506023 | 0.316317284 | down |
| TRINITY_DN37812_c0_g1 | Antithrombin-III | SERPINC1 | -5.176638235 | 0.014440153 | down |
| TRINITY_DN17042_c0_g1 | Tissue-type plasminogen activator | PLAT | -1.207714902 | 0.11798013 | down |
| TRINITY_DN29597_c0_g1 | Complement factor H | CFH | -3.84647223 | 0.292100182 | down |
| TRINITY_DN16571_c0_g3 | Prothrombin | F2 | -4.470230696 | 0.11137573 | down |
| TRINITY_DN32620_c0_g1 | Complement factor B | CFB | -1.190587141 | 0.105883638 | down |
| TRINITY_DN15614_c0_g1 | Fibrinogen beta chain | FGB | -3.540571126 | 0.000410863 | down |
| TRINITY_DN8167_c0_g1 | Fibrinogen alpha chain | FGA | -3.161983112 | 1.46072E-09 | down |
| TRINITY_DN20005_c0_g1 | Vitronectin | VTN | -2.859603864 | 0.025588603 | down |
| TRINITY_DN12291_c0_g1 | Fibrinogen gamma chain | FGG | -3.972023644 | 1.77296E-11 | down |
| TRINITY_DN7824_c1_g1 | Complement C3 (Fragment) | C3 | -2.253009175 | 0.000126314 | down |

**Table S11. Representative autophagy-animals differentiatio-related DEGs.**

| Gene ID | Description | Gene name | log2 Fold Change | Padj | Regulation |
|---|---|---|---|---|---|
| TRINITY_DN34414_c0_g1 | Cysteine protease ATG4B | ATG4B | 1.467484798 | 0.008066613 | up |
| TRINITY_DN3392_c0_g1 | Lysosome-associated membrane glycoprotein 1 | LAMP1 | 1.036637233 | 2.02688E-08 | up |
| TRINITY_DN30701_c0_g1 | GTPase HRas | HRAS | 1.976954019 | 0.040414067 | up |
| TRINITY_DN9426_c0_g1 | Ras-related protein Rab-33B | RAB33B | 1.507267112 | 1.38884E-06 | up |
| TRINITY_DN548_c1_g2 | GTP-binding protein Rheb | RHEB | 1.087540797 | 1.45318E-06 | up |
| TRINITY_DN14332_c0_g1 | Serine/threonine-protein kinase TBK1 | TBK1 | 1.350396008 | 1.47076E-05 | up |
| TRINITY_DN7530_c1_g1 | Autophagy-related protein 2 homolog B | ATG2B | 1.384410189 | 4.765E-07 | up |
| TRINITY_DN11424_c0_g1 | Autophagy-related protein 2 homolog A | ATG2A | 1.015800141 | 0.001068409 | up |
| TRINITY_DN512_c1_g1 | Serine/threonine-protein kinase ULK1 | ULK1 | 2.538192674 | 4.04697E-30 | up |
| TRINITY_DN512_c0_g1 | Serine/threonine-protein kinase ULK2 | ULK2 | 1.47394921 | 1.06579E-13 | up |
| TRINITY_DN10690_c0_g1 | 3-phosphoinositide-dependent protein kinase 1 | PDPK1 | 2.291186344 | 0.001523241 | up |
| TRINITY_DN9451_c0_g1 | Phosphatidylinositol 4,5-bisphosphate 3-kinase catalytic subunit beta isoform | PIK3CB | 1.23611094 | 0.275746434 | up |
| TRINITY_DN6559_c0_g1 | finger FYVE domain-containing protein 1 | ZFYVE1 | 1.714175721 | 8.23391E-12 | up |
| TRINITY_DN16315_c0_g2 | Endophilin-B2 | SH3GLB2 | 1.181044435 | 0.346379332 | up |
| TRINITY_DN1701_c0_g1 | Autophagy-related protein 9A | ATG9A | 1.49778288 | 1.2785E-10 | up |
| TRINITY_DN1946_c0_g1 | Insulin receptor substrate 2 | IRS2 | 1.222588373 | 1.71359E-08 | up |
| TRINITY_DN20_c2_g1 | Ras-related protein Rab-1A | RAB1A | 1.029786561 | 1.90819E-06 | up |
| TRINITY_DN3237_c0_g1 | Death-associated protein kinase 2 | DAPK2 | 1.110805911 | 0.248865636 | up |
| TRINITY_DN926_c0_g2 | RAC-beta serine/threonine-protein kinase | AKT2 | -1.330040334 | 7.05646E-10 | down |
| TRINITY_DN11826_c0_g1 | Insulin receptor substrate 2-A | IRS2-A | -1.523704072 | 3.69168E-08 | down |
| TRINITY_DN557_c4_g1 | Apoptosis regulator Bcl-2 | BCL2 | -1.30984671 | 0.001487134 | down |

| | | | | | |
|---|---|---|---|---|---|
| TRINITY_DN5141_c0_g3 | Ribosomal protein S6 kinase beta-1 | RPS6KB1 | -1.098043137 | 0.059707201 | down |
| TRINITY_DN18882_c1_g1 | RAC-alpha serine/threonine-protein kinase | AKT1 | -1.135963714 | 0.026267496 | down |
| TRINITY_DN3441_c0_g1 | Mitogen-activated protein kinase 8B | MAPK8B | -1.219241516 | 0.000897385 | down |
| TRINITY_DN16509_c0_g1 | Lysosome-associated membrane glycoprotein 1 | LAMP1 | -3.736830291 | 0.255351522 | down |
| TRINITY_DN28149_c0_g2 | Protein kinase C theta type | PRKCQ | -1.117659901 | 0.002384894 | down |
| TRINITY_DN2958_c0_g1 | Insulin receptor substrate 1-B | IRS1-B | -2.435894939 | 1.80999E-24 | down |
| TRINITY_DN8743_c0_g1 | GTPase KRas OS=Xenopus laevis | KRAS | -1.027761902 | 0.01644344 | down |
| TRINITY_DN10648_c0_g1 | Inositol 1,4,5-trisphosphate receptor type 1 | ITPR1 | -1.138208149 | 0.234924186 | down |

23

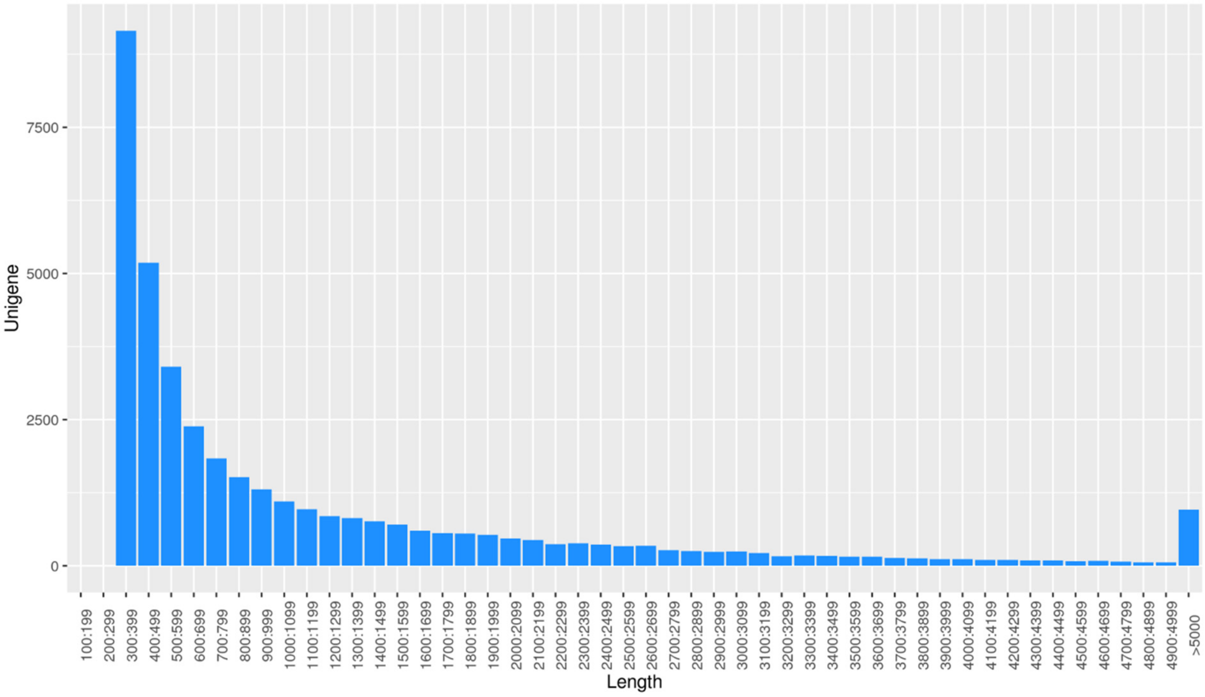


**Figure S1. The information of de novo assembly unigenes.**

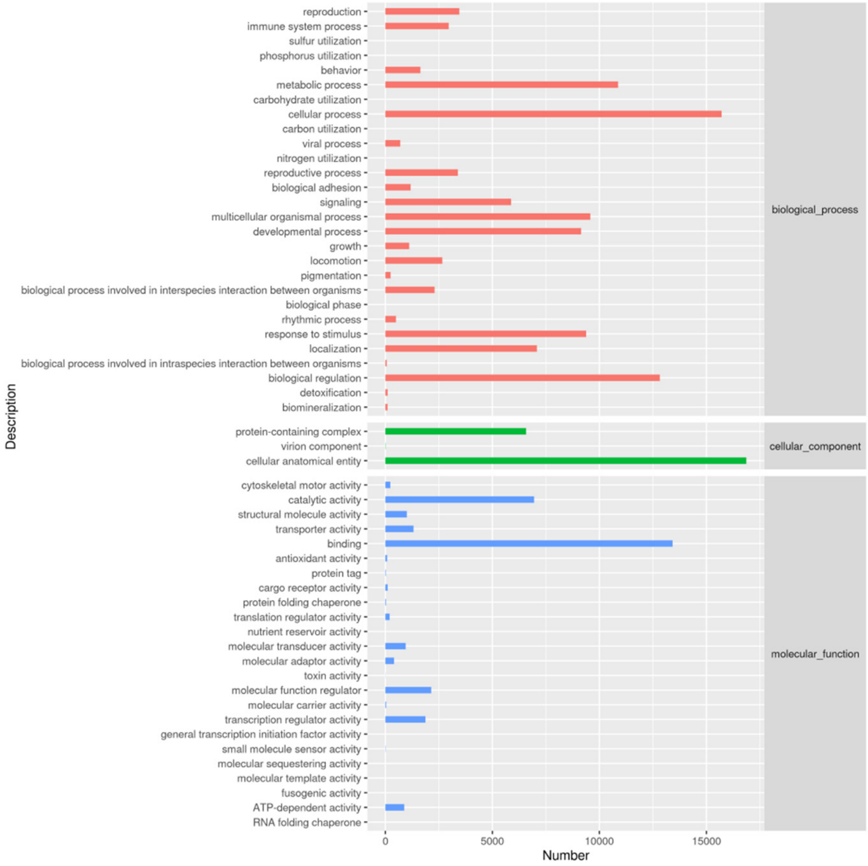

**Figure S2. GO annotations of the assembled unigenes of hybrid species.**

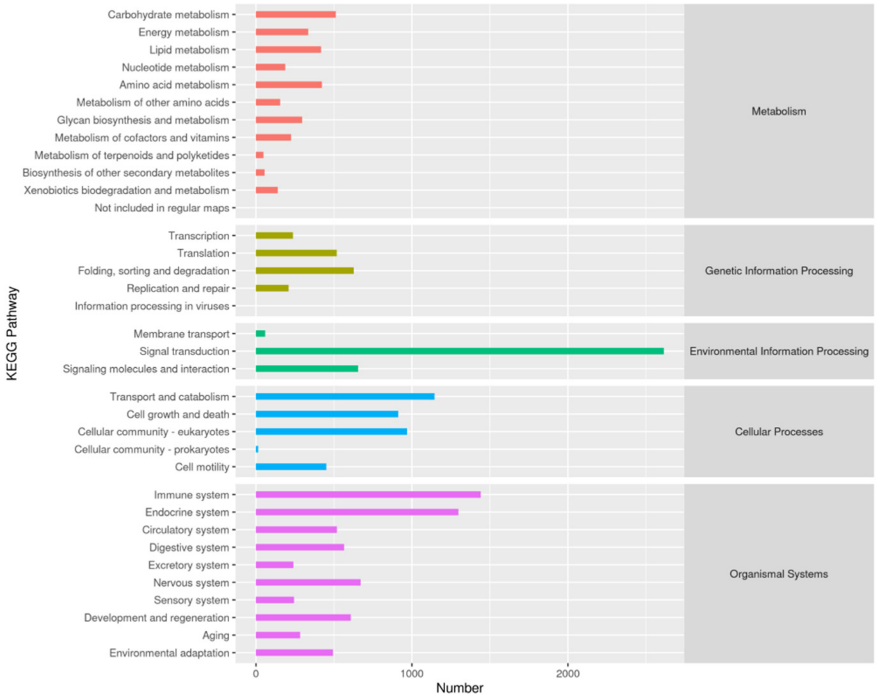


**Figure S3. KEGG annotations of the assembled unigenes of hybrid species.**

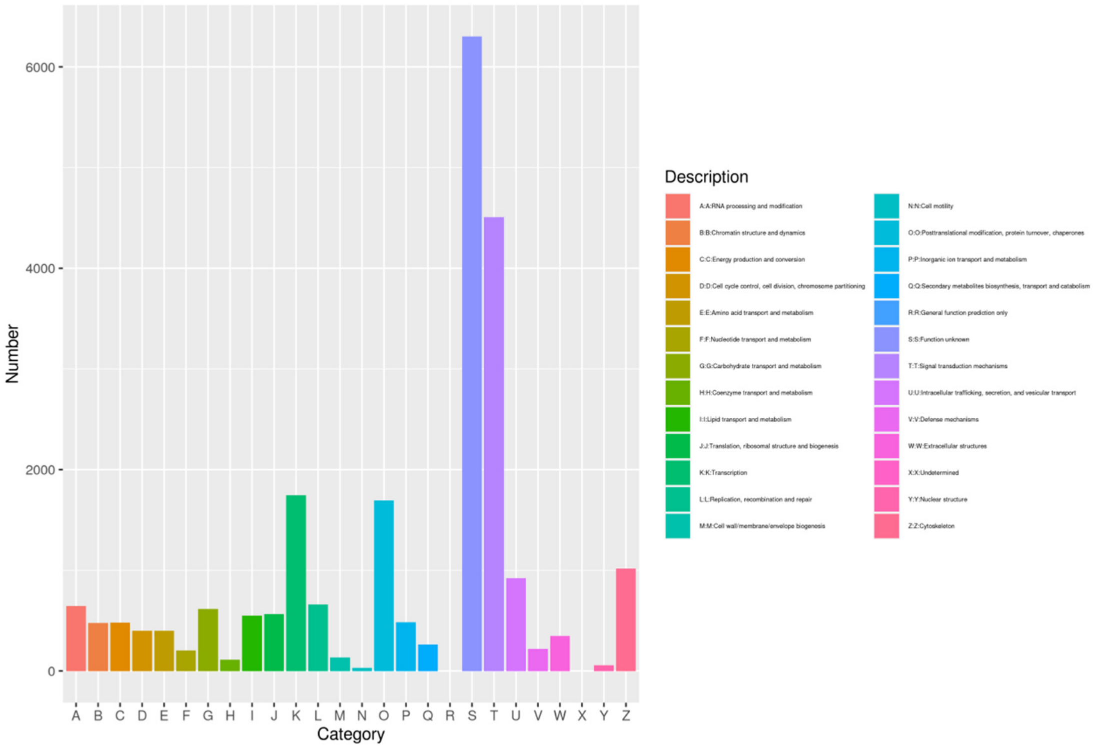

**Figure S4. EggNOG annotations of the assembled unigenes of hybrid species.**

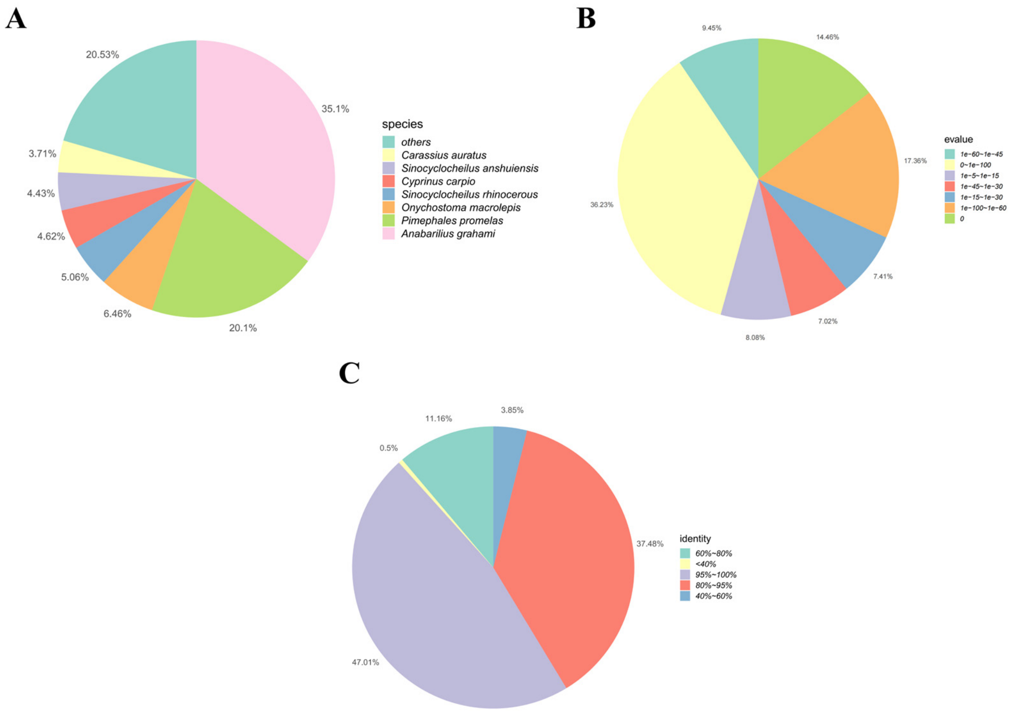


**Figure S5. NR annotations of the assembled unigenes of hybrid species.** (A) Distribution of species. (B) E-value distribution of comparisons. (C) Sequence similarity distribution.
